# Photodynamic Therapy Is Effective Against *Candida auris* Biofilms

**DOI:** 10.3389/fcimb.2021.713092

**Published:** 2021-09-03

**Authors:** Priyanka S. Bapat, Clarissa J. Nobile

**Affiliations:** ^1^Department of Molecular and Cell Biology, School of Natural Sciences, University of California Merced, Merced, CA, United States; ^2^Quantitative and Systems Biology Graduate Program, University of California Merced, Merced, CA, United States; ^3^Health Sciences Research Institute, University of California Merced, Merced, CA, United States

**Keywords:** *Candida auris* biofilms, drug resistance, red, green and blue (RGB) visible lights, photodynamic therapy, photosensitizing compounds, reactive oxygen species (ROS), non-drug therapeutic strategies, non-drug antifungal strategies

## Abstract

Fungal infections are increasing in prevalence worldwide. The paucity of available antifungal drug classes, combined with the increased occurrence of multidrug resistance in fungi, has led to new clinical challenges in the treatment of fungal infections. *Candida auris* is a recently emerged multidrug resistant human fungal pathogen that has become a worldwide public health threat. *C. auris* clinical isolates are often resistant to one or more antifungal drug classes, and thus, there is a high unmet medical need for the development of new therapeutic strategies effective against *C. auris*. Additionally, *C. auris* possesses several virulence traits, including the ability to form biofilms, further contributing to its drug resistance, and complicating the treatment of *C. auris* infections. Here we assessed red, green, and blue visible lights alone and in combination with photosensitizing compounds for their efficacies against *C. auris* biofilms. We found that (1) blue light inhibited and disrupted *C. auris* biofilms on its own and that the addition of photosensitizing compounds improved its antibiofilm potential; (2) red light inhibited and disrupted *C. auris* biofilms, but only in combination with photosensitizing compounds; and (3) green light inhibited *C. auris* biofilms in combination with photosensitizing compounds, but had no effects on disrupting *C. auris* biofilms. Taken together, our findings suggest that photodynamic therapy could be an effective non-drug therapeutic strategy against multidrug resistant *C. auris* biofilm infections.

## Introduction

Fungi are responsible for a wide range of infections in humans, including superficial skin infections as well as life-threatening disseminated infections (Brown et al., 2012). Three major classes of antifungal drugs (the polyenes, azoles, and echinocandins) are the most commonly used therapeutic agents for treating invasive fungal infections in humans (Odds et al., 2003; Prasad et al., 2016). The azoles and polyenes target the fungal cell membrane, while echinocandins target the fungal cell wall; thus, there is a need for new antifungal strategies with distinct mechanisms of action (Odds et al., 2003; Prasad et al., 2016).

*Candida auris* is a recently emerged human fungal pathogen belonging to the *Candida/Clavispora* clade that was first isolated from the ear canal of a patient in Japan in 2009, and has since been identified in over 35 countries (Satoh et al., 2009; Saris et al., 2018). *C. auris* is highly transmissible through surface contact, and has been isolated from the surfaces of windows, floors, curtains, bedrails, monitors, and other surfaces in healthcare settings (Welsh et al., 2017; Adams et al., 2018; Horton and Nett, 2020). In infected patients, *C. auris* is typically isolated from the skin, nares, wounds, axilla, and urinary tracts, as well as the bloodstream, bones, and cerebrospinal fluids of patients with severe invasive infections (Borman et al., 2016; Calvo et al., 2016; Morales-López et al., 2017; Horton and Nett, 2020). Once *C. auris* infections become systemic, they are associated with high mortality rates, ranging from 30-72%, with the highest mortality rates reported in patients with histories of extended hospital stays, implanted medical devices, or patients who have previously been treated with antifungal drugs (Cortegiani et al., 2018; Osei Sekyere, 2018; Spivak and Hanson, 2018; Chakrabarti and Singh, 2020; Garcia-Bustos et al., 2020; Shastri et al., 2020).

Since its emergence in 2009, *C. auris* clinical isolates have been reported to be resistant to one or more of the three major classes of antifungal drugs used to treat invasive fungal infections, with 90% resistant to at least one antifungal drug class, 30% resistant to at least two antifungal drug classes, and a handful displaying pan-resistance to all three major antifungal drug classes (Lockhart et al., 2017; Eyre et al., 2018; Cortegiani et al., 2019; Forsberg et al., 2019; Chakrabarti and Singh, 2020). *C. auris* resistance mechanisms are multifactorial, and have been reported to include the overexpression of the major facilitator superfamily (MFS) and ATP-binding cassette (ABC) drug efflux pumps, mutations in the ergosterol biosynthesis pathway, such as in the *ERG11* gene, and mutations in the *FKS1* gene, encoding a glucan synthase (Cortegiani et al., 2018; Kean et al., 2018; Chaabane et al., 2019; Dominguez et al., 2019; Bravo Ruiz and Lorenz, 2021). Given its heightened drug resistance and transmissibility, *C. auris* has become a serious global health threat (Lockhart et al., 2017; Piedrahita et al., 2017; Chaabane et al., 2019).

In the current coronavirus disease 2019 (COVID-19) pandemic, coinfections of *C. auris* with severe acute respiratory syndrome coronavirus 2 (SARS-CoV-2), have been increasingly reported, with high mortality rates (~60%), especially for critically ill patients who remain in the hospital for extended periods of time (>20 days) and in patients with implanted medical devices (e.g., catheters and ventilators) (Chowdhary et al., 2020; Steele et al., 2020; Zuo et al., 2020; Villanueva-Lozano et al., 2021). Additionally, high mortality rates (50-60%), have also been reported for *C. auris-*SARS-CoV-2 coinfections in patients with underlying chronic conditions, such as diabetes mellitus and kidney disease (Chowdhary et al., 2020; Rodriguez et al., 2020; Allaw et al., 2021; De Almeida et al., 2021; Magnasco et al., 2021; Prestel et al., 2021). The increased spread of *C. auris* infections during the COVID-19 pandemic is likely facilitated, at least in part, by the transformation of intensive care units and other hospital facilities into dedicated COVID-19 units, which foster ideal conditions for *C. auris* outbreaks (Chowdhary and Sharma, 2020; Villanueva-Lozano et al., 2021).

**Figure 1:**
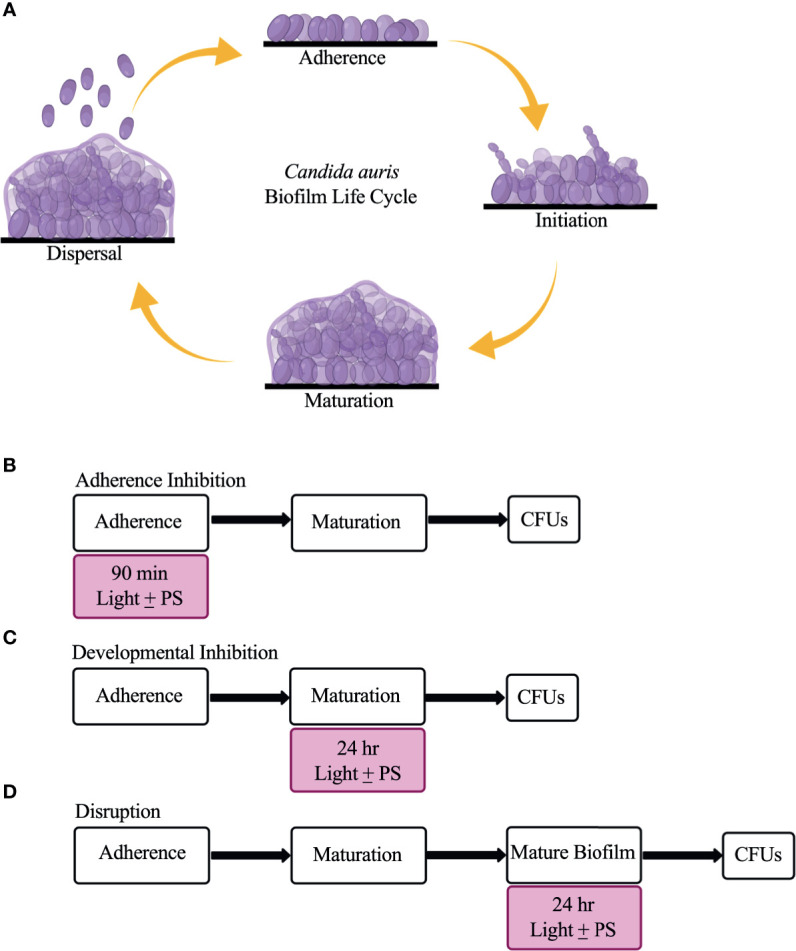
The *C*. *auris* biofilm life cycle and the three biofilm assays used in this study to assess the effectiveness of visible lights with and without photosensitizing compounds. **(A)** The *C*. *auris* biofilm life cycle occurs in four stages: adherence, initiation, maturation, and dispersal. During the adherence stage, planktonic *C*. *auris* yeast-form cells adhere to a surface. During the initiation stage, the adhered cells proliferate, and some pseudohyphal cells are formed. During the maturation stage, the cells continue to proliferate and an extracellular matrix composed of glucans and mannans encases the biofilm cells. Finally, in the dispersal stage, yeast-form cells leave the biofilm to adhere to and form biofilms on new surfaces, or enter the bloodstream to cause systemic infections. **(B)** Overview of the adherence inhibition biofilm assay, where the visible light with (+) and without (-) the photosensitizing compound (PS) were present during the 90-min adherence stage of biofilm formation. **(C)** Overview of the developmental inhibition biofilm assay, where the visible light with (+) and without (-) the PS were present during the 24-h maturation stage of biofilm formation. **(D)** Overview of the disruption biofilm assay, where the visible light with (+) and without (-) the PS were present for an additional 24 h on a mature (24-h) biofilm. CFUs were measured to determine viable cell counts at the end of each biofilm assay. This figure was created using BioRender.com, and adapted from Bapat et al., 2021.

*C. auris* possesses multiple virulence traits that contribute to its pathogenicity, including the formation of biofilms (Forsberg et al., 2019; Chakrabarti and Singh, 2020). Biofilms are defined as communities of adherent microbial cells encased in a protective extracellular matrix (Kolter and Greenberg, 2006; López et al., 2010). *C. auris* biofilms are composed primarily of yeast-form cells interspersed with pseudohyphal cells that are encased in a mannan and glucan extracellular matrix (Sherry et al., 2017; Dominguez et al., 2019; Romera et al., 2019). Although planktonic *C. auris* cells display antifungal drug resistance on their own, *C. auris* cells isolated from biofilms are even more resistant to antifungal drugs than their free-floating counterparts (Larkin et al., 2017; Sherry et al., 2017; Cortegiani et al., 2018; Horton and Nett, 2020). *C. auris* biofilm formation is thought to occur in four stages: adherence, initiation, maturation, and dispersal (Kean et al., 2018; Dominguez et al., 2019) (**Figure 1A**). In the adherence stage, planktonic *C. auris* yeast-form cells adhere to biotic surfaces (e.g., skin, and mucosal layers) or abiotic surfaces (e.g., catheters, and prosthetic joints). In the initiation stage, the adhered *C. auris* yeast-form cells begin to proliferate, and some pseudohyphal cells develop. In the maturation stage, the cells within the *C. auris* biofilm continue to proliferate and an extracellular matrix that encases the biofilm cells is formed. Finally, in the dispersal stage, some *C. auris* yeast-form cells exit the biofilm to adhere to and form biofilms on new surfaces or enter the bloodstream to cause systemic infections.

Given that *C. auris* clinical isolates are often resistant to one or more antifungal drug classes, there is a high unmet medical need for the development of new therapeutic strategies effective against *C. auris*. Photodynamic therapy has been used for the past 40 years to treat oncologic skin conditions, and more recently to treat benign inflammatory skin conditions, such as acne vulgaris and viral warts (Agostinis et al., 2011; Kim et al., 2015; Cohen et al., 2016). It has also been gaining scientific interest as a non-drug therapeutic strategy to treat a variety of infections (Cieplik et al., 2018). Photodynamic therapy requires the presence of a light source, a non-toxic photosensitizing compound, and molecular oxygen (Wainwright et al., 1998; Hamblin and Hasan, 2004; Wainwright et al., 2017). Following light exposure and absorption, the photosensitizing compound transfers electrons to molecular oxygen, which acts as an electron acceptor, ultimately leading to the production of cytotoxic reactive oxygen species (ROS), such as singlet oxygen, hydroxyl radicals, and superoxide anions (St. Denis et al., 2011; Lyon et al., 2011; Vatansever et al., 2013; Wainwright et al., 2017). Unlike traditional antimicrobial drugs, photodynamic therapy affects numerous microbial targets simultaneously, making it unlikely for resistance to develop. Given its non-specific mechanisms of action, photodynamic therapy could be a clinically useful therapeutic strategy effective against a multitude of infections, including those caused by multidrug resistant *C. auris*.

Broadly, the visible light spectrum is divided into red (620-700 nm), green (500-560 nm), and blue (400-490 nm) wavelengths, of which certain discreet wavelengths have been reported to display antimicrobial properties (Bruno and Svoronos, 2006; Denis et al., 2011; Wainwright et al., 2017; Cieplik et al., 2018; Gwynne and Gallagher, 2018). Blue light has been shown to effectively kill several different species of pathogenic bacteria and fungi, including methicillin resistant *Staphylococcus aureus*, carbapenem resistant *Klebsiella pneumoniae*, and ß-lactam resistant *Escherichia coli* (Dai et al., 2013; Murdoch et al., 2013; Cieplik et al., 2014; Teixeira et al., 2015; Halstead et al., 2016; Moorhead et al., 2016; Zhang et al., 2016; Dai, 2017; Guffey et al., 2017; Trzaska et al., 2017; Wang et al., 2017; Pinto et al., 2018; Wang et al., 2018; Ferrer-Espada et al., 2019). Compared to blue light, the antimicrobial properties of red and green lights have been considerably less studied to date (Vural et al., 2008; Huh et al., 2012; Gwynne and Gallagher, 2018; Panariello et al., 2019).

Although it has been shown that visible lights can have antimicrobial effects on their own, likely by generating ROS *via* the photoexcitation of naturally occurring photosensitizing compounds, such as porphyrins, the antimicrobial effects of visible lights combined with exogenous synthetic photosensitizing compounds have been shown to substantially increase the generation of ROS (Wainwright et al., 1998; Carvalho et al., 2009; Cieplik et al., 2014; Romano et al., 2017). Recently, the antimicrobial effects of visible lights alone and in combination with classic photosensitizing compounds, were comprehensively assessed against *Candida albicans* biofilms (Bapat et al., 2021). In this study, blue light was found to be highly effective at inhibiting and disrupting *C. albicans* biofilms on its own and the addition of photosensitizing compounds increased its antibiofilm effectiveness, while red and green lights were found to inhibit *C. albicans* biofilm formation only in combination with photosensitizing compounds, but were unable to disrupt biofilms. In terms of *C. auris*, to our knowledge, only one study to date has assessed the effects of photodynamic therapy on *C. auris* biofilms. In this study, red light combined with the photosensitizing compound methylene blue was found to be highly effective at reducing viable cell counts from *C. auris* biofilms (Tan et al., 2019).

To better understand the benefits of photodynamic therapy against *C. auris* infections, here we comprehensively assessed the efficacies of red, green, and blue visible lights alone and in combination with the classic photosensitizing compounds new methylene blue, toluidine blue O, and rose bengal, against *C. auris* biofilms. We found that blue light inhibited and disrupted *C. auris* biofilms on its own, and that the addition of photosensitizing compounds improved its antibiofilm effectiveness. We found that red light inhibited and disrupted *C. auris* biofilms, but only in combination with photosensitizing compounds. Finally, we found that green light inhibited *C. auris* biofilms in combination with photosensitizing compounds, but had no effects on disrupting *C. auris* biofilms. In general, the effects we observed on *C. auris* biofilms were similar across biofilms formed by different *C. auris* clinical isolates from distinct genetic clades that display different antifungal drug susceptibilities.

## Materials and Methods

### Strains and Media

Given that the effects of visible lights on *C. albicans* biofilms have been comprehensively assessed (Bapat et al., 2021), we used the *C. albicans* clinical isolate SC5314 (Meyers et al., 1968) as a reference strain in this study. We used the following *C. auris* clinical isolates: Strain #0383 (AR0383; South African clade), Strain #0389 (AR0389; South Asian clade), and Strain #0390 (AR0390; South Asian clade) (Centers for Disease Control and Prevention (CDC) AR Isolate Bank, Drug Resistance *Candida* species panel; https://wwwn.cdc.gov/ARIsolateBank/; accessed on 02/20/2021). The minimum inhibitory concentrations (MICs) for representative drugs from the three major antifungal drug classes used to treat invasive fungal infections for each *C. auris* isolate used in this study have been reported previously (Lockhart et al., 2017; https://www.cdc.gov/fungal/candida-auris/c-auris-antifungal.html/; accessed on 05/07/2021), and can be found in **Table S1**. *C. auris* and *C. albicans* cells were recovered from -80°C glycerol stocks for two days at 30°C on yeast extract peptone dextrose (YPD) agar plates [1% yeast extract (Thermo Fisher Scientific, Catalog #211929), 2% Bacto peptone (Gibco, Catalog #211677), 2% dextrose (Fisher Scientific Catalog #D16-3), and 2% agar (Criterion, Catalog #89405-066)]. Overnight cultures were grown for ~15 h at 30°C, shaking at 225 rpm in YPD liquid medium [1% yeast extract (Thermo Fisher Scientific, Catalog #211929), 2% Bacto peptone (Gibco, Catalog #211677), and 2% dextrose (Fisher Scientific Catalog #D16-3)]. All biofilm assays were performed using RPMI-1640 medium with L-glutamine and without sodium bicarbonate (Sigma Aldrich, Catalog #R6504-10X1L), supplemented with 34.5 g/L MOPS (Sigma Aldrich, Catalog #M3183), adjusted to pH 7.2 with sodium hydroxide (Fisher Scientific, Catalog #S318-100), and filter sterilized using a 0.22 µm filter (Corning, Catalog #431098).

### Light Sources and Photosensitizing Compounds

A red LED light source (ABI LED lighting, Catalog #GR-PAR38-26W-RED, 26-Watt 620-630 nm, outputting 176 J/cm^2^), a green LED light source (ABI LED lighting, #GR-PAR38-24W-520nm, 24-Watt 520-530 nm, outputting 204 J/cm^2^), and a blue LED light source (ABI LED lighting, GR-PAR38-24W-BLU, 24-Watt 450 nm, outputting 240 J/cm^2^) were placed at a distance of 8 inches directly above the biofilm wells and were used as described previously in the biofilm assays (Bapat et al., 2021). Average LED light intensity measurements at this distance were 6500 lux for red light, 6700 lux for green light, and 5900 lux for blue light.

The photosensitizing compounds new methylene blue (Sigma Aldrich, Catalog #B-4631), toluidine blue O (Sigma Aldrich, Catalog #T3260), and rose bengal (Sigma Aldrich, Catalog #198250) were added alone and in combination with the red, green, and blue visible lights in the biofilm assays. The photosensitizing compounds were dissolved in PBS (HyClone, Catalog #16777-252) at a stock concentration of 10 mM and diluted to a working concentration of 400 μM in RPMI-1640 medium, which was used to grow the biofilms. Stocks of the photosensitizing compounds were prepared fresh every two weeks, filter sterilized using a 0.22 µm filter, and stored at 4°C in the dark.

### Biofilm Assays

The adherence inhibition, developmental inhibition, and disruption biofilm assays were performed as described previously (Bapat et al., 2021), where colony forming units (CFUs) were measured at the end of the assays to assess the efficacies of the visible lights with or without photosensitizing compounds at reducing *C. auris* and *C. albicans* viable cell counts from the biofilms.

Briefly, biofilms were grown in triplicate on the bottoms of sterile flat-bottomed 12-well non-tissue culture treated polystyrene plates (Corning, Catalog #351143). The 12-well plates were seeded with *Candida* cells at a final OD_600_ of 0.5 in a final volume of 2 mL of RPMI-1640 medium and grown for 90 min at 37°C, with shaking at 250 rpm in an ELMI shaker (M2 Scientifics, Catalog #ELMI-TRMS 04). After the 90-min adherence stage, the wells were washed gently with PBS and fresh RPMI-1640 medium was added to each well. The plates were sealed with breathable sealing membranes (Sigma Aldrich, Catalog #Z380059) and grown for 24 h at 37°C, with shaking at 250 rpm in an ELMI shaker. For the adherence inhibition biofilm assay, the biofilms were exposed to red, green, or blue visible lights with or without a photosensitizing compound during the 90-min adherence stage of biofilm formation (**Figure 1B**). For the developmental inhibition biofilm assay, the biofilms were exposed to red, green, or blue visible lights with or without a photosensitizing compound throughout the first 24 h of biofilm growth, but not during the initial 90-min adherence stage (**Figure 1C**). For the disruption biofilm assay, biofilms were grown, medium was removed from each well containing mature 24-h biofilms, fresh RPMI-1640 medium was added to each well, the plates were re-sealed, and the mature biofilms were exposed to red, green, or blue visible lights with or without a photosensitizing compound for an additional 24 h (**Figure 1D**). The 12-well plates were divided such that half of one plate was exposed to the light of interest and the other half was covered with foil and served as a no light control.

### Determination of Colony Forming Units From *Candida* Biofilms

CFU determinations from biofilms were performed as previously described (Lohse et al., 2017; Gulati et al., 2018; Bapat et al., 2021). Briefly, biofilms were scraped from the bottoms of the each well of a 12-well plate using a sterile spatula, vigorously vortexed, serially diluted in PBS, and plated onto YPD agar plates. The plates were incubated at 30°C for 2 days and colonies were counted to determine CFUs/mL. Statistical significance was determined using Student’s unpaired two-tailed t-tests assuming unequal variance.

### Viability Staining of *C. auris* Biofilms

To assess the viability of *C. auris* biofilm cells, viability staining was performed both on *C. auris* biofilms directly and on *C. auris* cells resuspended from biofilms under each light and photosensitizing compound treatment condition using the LIVE/DEAD *Bac*Light viability kit (Invitrogen, Catalog #L7012) as described previously for use on *C. albicans* biofilms (Jin et al., 2005; Bapat et al., 2021), and according to the manufacturer’s protocol. Briefly, the samples were incubated with 3 μL of SYTO9 and 3 μL of propidium iodide in the dark at 30°C for 20 min. Following incubation, the samples were imaged by fluorescence microscopy at 20X magnification with a green laser (GFP/green channel; 470 nm excitation wavelength) and a red laser (Texas Red/red channel; 585 nm excitation wavelength) using an EVOS Cell Imaging System (Life Technologies, Catalog #EVOS FL Cell Imaging System).

## Results

### Blue Visible Light Alone Is Effective Against *C. auris* Biofilms

**Figure 2:**
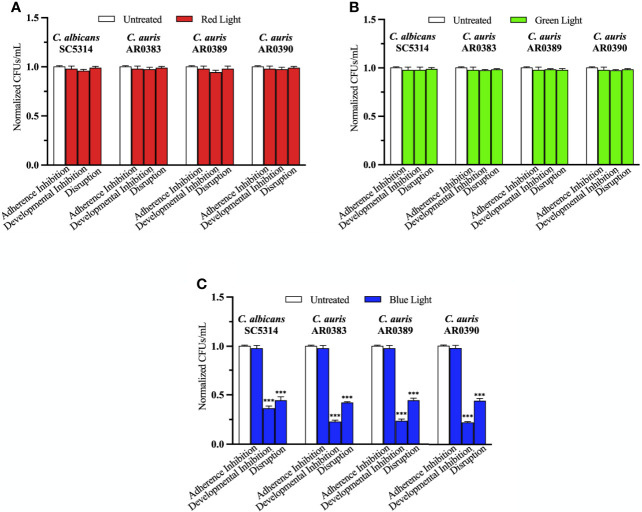
Blue visible light alone is effective against *C*. *auris* biofilms. *C*. *albicans* (SC5314) and *C*. *auris* (AR0383, AR0389, and AR0390) biofilms were exposed to red, green, and blue visible lights individually in the adherence inhibition, developmental inhibition, and disruption biofilm assays. CFUs/mL were counted to determine viable cell counts at the end of each of the biofilm assays. Effects of **(A)** red light alone (Red Light), **(B)** green light alone (Green Light), and **(C)** blue light alone (Blue Light) in the three different biofilm assays compared to an untreated control (Untreated). Standard deviations are shown for each sample (n = 3). The average CFUs/mL of the untreated control samples for each assay were normalized to 1. Significance comparisons are relative to the untreated control and were determined using student’s unpaired two-tailed t-tests assuming unequal variance for *p* ≤ 0.001 (***).

To determine whether red, green, and blue visible lights on their own can affect *C. auris* biofilm development, we first performed the three biofilm assays in the presence of each of these visible light treatments. We used three *C. auris* clinical isolates encompassing two different genetic clades (AR0383 from the South African clade, AR0389 from the South Asian clade, and AR0390 from the South Asian clade). We found that red and green visible lights on their own had no effects on *C. auris* biofilms in any of the three biofilm assays compared to the untreated control (**Figures 2A, B**). We also found that blue light on its own had no effect at inhibiting *C. auris* biofilm formation in the adherence inhibition biofilm assay compared to the untreated control (**Figure 2C**). However, blue light on its own was effective at inhibiting *C. auris* biofilm formation by 77% (averaging all three *C. auris* strains) in the developmental inhibition biofilm assay (*p=*0.0001) (**Figure 2C**). We also found that blue light on its own was effective at disrupting *C. auris* biofilms by 57% (averaging all three *C. auris* strains) in the disruption biofilm assay (*p=*0.0004) (**Figure 2C**).

### Red, Green, and Blue Visible Lights in Combination With Photosensitizing Compounds Are Effective Against *C. auris* Biofilms

**Figure 3:**
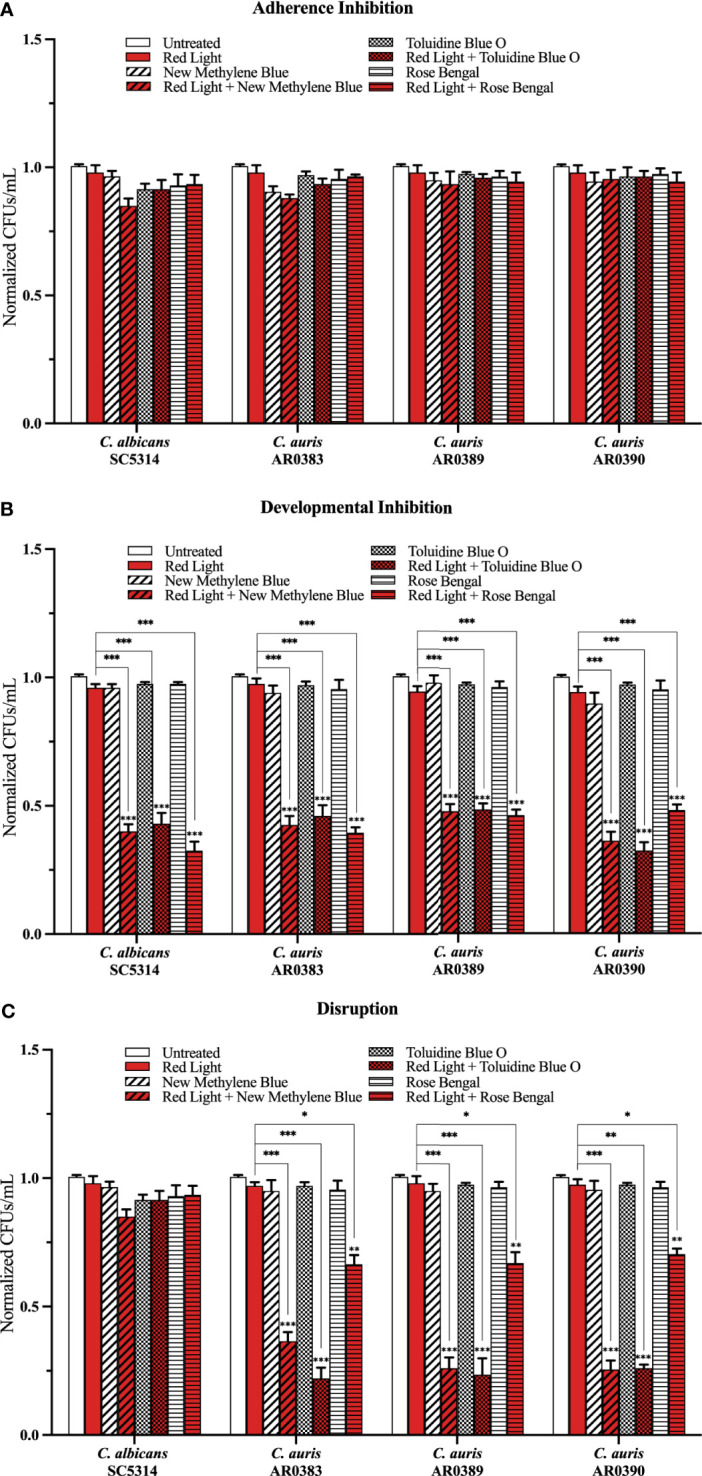
Red visible light in combination with photosensitizing compounds is effective against *C*. *auris* biofilms. *C albicans* (SC5314) and *C*. *auris* (AR0383, AR0389, and AR0390) biofilms were exposed to red visible light with and without the photosensitizing compound indicated in the **(A)** adherence inhibition, **(B)** developmental inhibition, and **(C)** disruption biofilm assays. Untreated control (Untreated), red light alone (Red Light), photosensitizing compound alone (New Methylene Blue, Toluidine Blue O, and Rose Bengal), and red light in combination with the photosensitizing compound (Red Light + New Methylene Blue, Red Light + Toluidine Blue O, and Red Light + Rose Bengal) are shown. CFUs/mL were measured to determine viable cell counts from the biofilms at the end of each biofilm assay. Standard deviations are shown for each sample (n = 3). The average CFUs/mL of the untreated control samples for each assay were normalized to 1. Significance comparisons are relative to the untreated control unless otherwise noted with significance bars and were determined using student’s unpaired two-tailed t-tests assuming unequal variance for *p* ≤ 0.05 (*), *p* ≤ 0.01 (**), and *p* ≤ 0.001 (***).

To determine whether red, green, and blue visible lights combined with classic exogenous photosensitizing compounds can affect *C. auris* biofilm development, we performed the three biofilm assays in the presence of each of these visible light treatments plus new methylene blue, toluidine blue O, and rose bengal, and assessed the effects of this treatment on *C. auris* biofilms formed by AR0383, AR0389, and AR0390. Compared to the average of the untreated control, red light on its own, and each photosensitizing compound on its own (i.e., the three negative controls), we found that red light plus any of the photosensitizing compounds had no effect on *C. auris* biofilm formation in the adherence inhibition biofilm assay (**Figure 3A**). Compared to the average of the three negative controls, we found that red light plus any of the photosensitizing compounds was effective at inhibiting *C. auris* biofilm formation by 58% when combined with new methylene blue (*p=*0.0001), 58% when combined with toluidine blue O (*p=*0.0002), and 55% when combined with rose bengal (*p=*0.0001) (averaging all three *C. auris* strains) in the developmental inhibition biofilm assay (**Figure 3B**). Compared to the average of the three negative controls, we found that red light plus any of the photosensitizing compounds was effective at disrupting mature *C. auris* biofilms by 71% when combined with new methylene blue (*p=*0.0005), 76% when combined with toluidine blue O (*p=*0.0004), and 32% when combined with rose bengal (*p=*0.009) (averaging all three *C. auris* strains) in the disruption biofilm assay (**Figure 3C**).

**Figure 4:**
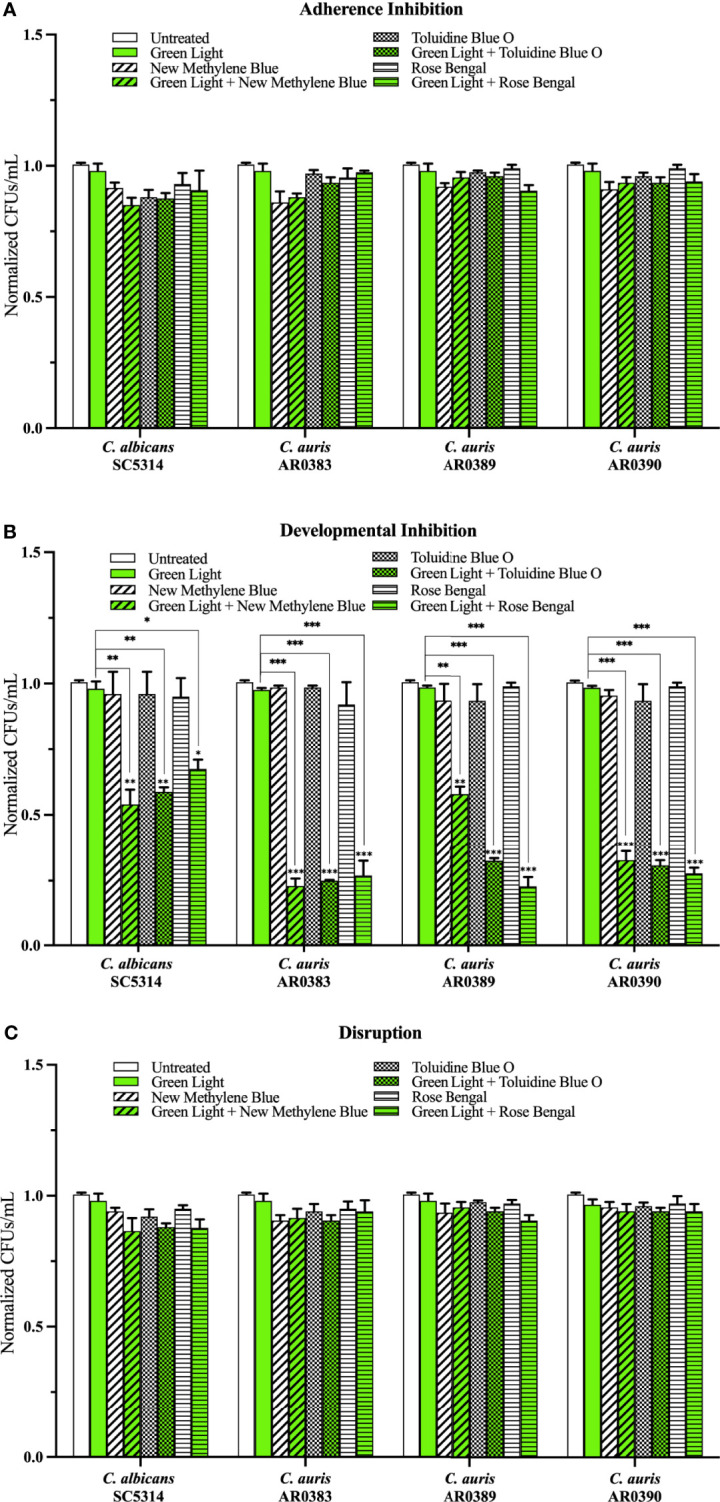
Green visible light in combination with photosensitizing compounds is effective against *C*. *auris* biofilms. *C albicans* (SC5314) and *C*. *auris* (AR0383, AR0389, and AR0390) biofilms were exposed to green visible light with and without the photosensitizing compound indicated in the **(A)** adherence inhibition, **(B)** developmental inhibition, and **(C)** disruption biofilm assays. Untreated control (Untreated), green light alone (Green Light), photosensitizing compound alone (New Methylene Blue, Toluidine Blue O, and Rose Bengal), and green light in combination with the photosensitizing compound (Green Light + New Methylene Blue, Green Light + Toluidine Blue O, and Green Light + Rose Bengal) are shown. CFUs/mL were counted to determine viable cell counts at the end of each of the biofilm assays. Standard deviations are shown for each sample (n = 3). The average CFUs/mL of the untreated control samples for each assay were normalized to 1. Significance comparisons are relative to the untreated control unless otherwise noted with significance bars and were determined using student’s unpaired two-tailed t-tests assuming unequal variance for *p* ≤ 0.05 (*), and *p* ≤ 0.01 (**) and *p* ≤ 0.001 (***).

Compared to the average of the untreated control, green light on its own, and each photosensitizing compound on its own (i.e., the three negative controls), we found that green light plus any of the photosensitizing compounds had no effect on *C. auris* biofilm formation in the adherence inhibition biofilm assay (**Figure 4A**). Compared to the average of the three negative controls, we found that green light plus any of the photosensitizing compounds was effective at inhibiting *C. auris* biofilm formation by 62% when combined with new methylene blue (*p=*0.004), 76% when combined with toluidine blue O (*p=*0.0007), and 74% when combined with rose bengal (*p=*0.0004) (averaging all three *C. auris* strains) in the developmental inhibition biofilm assay (**Figure 4B**). Compared to the average of the three negative controls, we found that green light plus any of the photosensitizing compounds was not effective at disrupting mature *C. auris* biofilms (averaging all three *C. auris* strains) in the disruption biofilm assay (**Figure 4C**).

**Figure 5:**
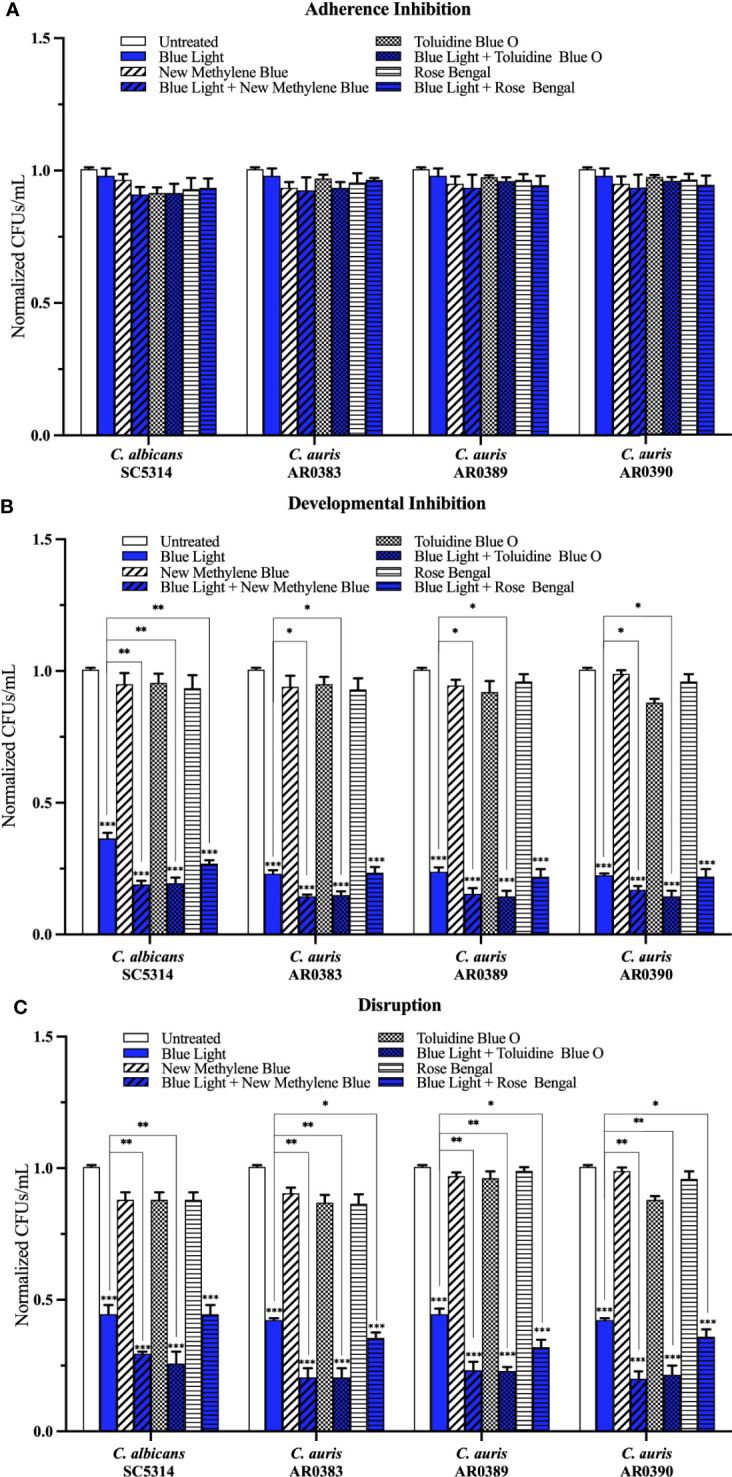
Blue visible light in combination with photosensitizing compounds is effective against *C*. *auris* biofilms. *C. albicans* (SC5314) and *C*. *auris* (AR0383, AR0389, and AR0390) biofilms were exposed to blue visible light with and without the photosensitizing compound indicated in the **(A)** adherence inhibition, **(B)** developmental inhibition, and **(C)** disruption biofilm assays. Untreated control (Untreated), blue light alone (Blue Light), photosensitizing compound alone (New Methylene Blue, Toluidine Blue O, and Rose Bengal), and blue light in combination with the photosensitizing compounds (Blue Light + New Methylene Blue, Blue Light + Toluidine Blue O, and Blue Light + Rose Bengal) are shown. CFUs/mL were counted to determine viable cell counts at the end of each of the biofilm assays. Standard deviations are shown for each sample (n = 3). The average CFUs/mL of the untreated control samples for each assay were normalized to 1. Significance comparisons are relative to the untreated control unless otherwise noted with significance bars and were determined using student’s unpaired two-tailed t-tests assuming unequal variance for *p* ≤ 0.05 (*), *p* ≤ 0.01 (**), and *p* ≤ 0.001 (***).

Compared to the average of the untreated control, blue light on its own, and each photosensitizing compound on its own, we found that blue light plus any of the three photosensitizing compounds had no effect on *C. auris* biofilm formation in the adherence inhibition biofilm assay (**Figure 5A**). Since blue light on its own was effective at inhibiting and disrupting *C. auris* biofilms in the developmental inhibition biofilm assay and the disruption biofilm assay, respectively (**Figure 2C**), we compared the effects of blue light plus the three photosensitizing compounds to the average of the untreated control and each photosensitizing compound on its own (i.e., the two negative controls) for these biofilm assays. Compared to the average of the two negative controls, we found that blue light plus any of the photosensitizing compounds was effective at inhibiting *C. auris* biofilm formation by 84% when combined with new methylene blue (*p=*0.00001), 85% when combined with toluidine blue O (*p=*0.00001), and 78% when combined with rose bengal (*p=*0.0001) (averaging all three *C. auris* strains) in the developmental inhibition biofilm assay (**Figure 5B**). Compared to the biofilm inhibitory effects of blue light on its own, we found that blue light plus new methylene blue had an additive inhibitory effect of 7% (*p*=0.01), and blue light plus toluidine blue O had an additive inhibitory effect of 8% (*p=*0.01) (averaging all three *C. auris* strains) in the developmental inhibition biofilm assay (**Figure 5B**). We did not observe an additive inhibitory effect of blue light plus rose bengal against *C. auris* biofilms in the developmental inhibition biofilm assay (**Figure 5B**). Compared to the average of the two negative controls, we found that blue light plus any of the photosensitizing compounds was effective at disrupting mature *C. auris* biofilms by 79% when combined with new methylene blue (*p=*0.0003), 79% when combined with toluidine blue O (*p=*0.0002), and 66% when combined with rose bengal (*p=*0.007) (averaging all three *C. auris* strains) in the disruption biofilm assay (**Figure 5C**). Compared to the biofilm disruption effects of blue light on its own, the combination of blue light plus new methylene blue had an additive biofilm disruption effect of 22% (*p=*0.002), blue light plus toluidine blue O had an additive effect of 22% (*p=*0.002), and blue light plus rose bengal had an additive effect of 9% (*p=*0.01) (averaging all three *C. auris* strains) in the disruption biofilm assay (**Figure 5C**).

Given that none of the light and photosensitizing compound combination treatments were effective at inhibiting *C. auris* biofilms in the adherence inhibition biofilm assay (90 min exposure), but were effective at inhibiting *C. auris* biofilms in the development inhibition biofilm assay (24 hr exposure) and were effective at disrupting *C. auris* biofilms in the disruption biofilm assay (24 hr exposure), we wondered whether these treatments would have antibiofilm effects on *C. auris* biofilms in the developmental inhibition and disruption biofilm assays if they were applied for only 90 min. To test this, we used blue light plus toluidine blue O, which was highly effective against *C. auris* biofilms, and assessed the effects of this treatment for only 90 min on *C. auris* biofilms formed by AR0383, AR0389, and AR0390 in the developmental inhibition and disruption biofilm assays. In this shortened timeframe (90 min exposure), we observed no significant antibiofilm effects in the developmental inhibition or disruption biofilm assays (**Figure S1**).

Finally, to independently assess cell viability within *C. auris* biofilms, we performed LIVE/DEAD staining assays on both *C. auris* biofilms directly and on *C. auris* cells resuspended from biofilms after treatment with the different visible lights and photosensitizing compounds. Our cell viability staining results were consistent with our CFU determinations for all treatment conditions (see **Figures S2–S6** for representative images from the LIVE/DEAD staining assays performed directly on *C. auris* biofilms formed by AR0383 and **Figures S7–S11** for representative images from the LIVE/DEAD staining assays performed on *C. auris* cells resuspended from biofilms formed by AR0383).

## Discussion

Photodynamic therapy is used today to treat oncological and inflammatory skin conditions; however, its use as an antimicrobial strategy is only beginning to be realized. Photodynamic therapy relies on the production of ROS that can have cytotoxic effects on targeted cells. To determine the utility of photodynamic therapy for use against *C. auris* infections, we assessed the antibiofilm effects of visible lights alone and in combination with classic photosensitizing compounds on *C*. *auris* biofilms. We found that, of the visible lights tested, blue light was the only visible light that had antibiofilm properties on its own against *C. auris* biofilms, where it clearly prevented biofilm formation when it was applied throughout biofilm development, as well as clearly disrupted biofilms when it was applied on a mature biofilm. Overall, we found that red, green, and blue visible lights when combined with photosensitizing compounds, prevented *C. auris* biofilm formation when applied throughout biofilm development; however, only red and blue lights in combination with photosensitizing compounds disrupted mature *C. auris* biofilms. Interestingly, none of the visible lights and photosensitizing compound combination treatments were effective at inhibiting *C. auris* biofilms during the 90-min adherence stage of biofilm formation, at inhibiting *C. auris* biofilm development when the exposure time was shortened to 90 min, or at disrupting mature *C. auris* biofilms when the exposure time was shortened to 90 min, highlighting the potential importance of exposure time in the antibiofilm efficacy of photodynamic therapy.

Our findings on *C. auris* biofilms indicate that photosensitizing compounds can sensitize *C. auris* biofilms to visible lights when applied during biofilm development (i.e., over the course of a 24-hr period). We found that the combination treatments of red and blue lights with the photosensitizing compounds had the most striking antibiofilm effects, where these treatments both prevented *C. auris* biofilm formation as well as disrupted mature *C. auris* biofilms, significantly above red and blue light treatments alone. These effects were most notable when red and blue lights were combined with new methylene blue and toluidine blue O, which are structurally similar (phenothiazinium salt) photosensitizing compounds. Although the detailed mechanisms of how photosensitizing compounds sensitize *C. auris* biofilms to light exposure are not understood, photosensitizing compounds are generally known to enhance the production of ROS (Cieplik et al., 2014; Abrahamse and Hamblin, 2016; Cieplik et al., 2018), which likely leads to cytotoxicity of *C. auris* biofilm cells. Overall, our findings demonstrate that blue light combined with toluidine blue O, followed by blue light combined with new methylene blue, red light combined with toluidine blue O, and then red light combined with new methylene blue, are the most effective photodynamic therapy treatment combinations against *C. auris* biofilms.

In general, the majority of our findings on the effects of visible lights combined with photosensitizing compounds on *C. auris* biofilms are consistent with the effects of these treatments on *C. albicans* biofilms (Bapat et al., 2021); however, there are two notable species-specific differences that we would like to point out. First, we found that red light in combination with photosensitizing compounds was effective at disrupting mature *C. auris* biofilms by 60% on average, while this treatment had no effect on *C. albicans* biofilms. Second, we found that green light in combination with toluidine blue O, and green light in combination with rose bengal, were on average more effective at preventing *C. auris* biofilm formation by 32% and 42%, respectively, than they were at preventing *C. albicans* biofilm formation. These observed species-specific differences in treatment efficacies suggest that photodynamic therapy may be overall more effective against *C. auris* biofilms than against *C. albicans* biofilms, which may, in part, be due to structural differences between *C. auris* and *C. albicans* biofilms. For example, *C. auris* biofilms are generally thinner than *C. albicans* biofilms, and are composed of yeast-form cells with occasional pseudohyphal cells that are encased in a glucan and mannan extracellular matrix (Sherry et al., 2017; Dominguez et al., 2019; Romera et al., 2019). *C. albicans* biofilms, on the other hand, are generally thicker than *C. auris* biofilms, and are composed of yeast-form cells, pseudohyphal cells, and hyphal cells, encased in an extracellular matrix composed of proteins, lipids, carbohydrates, and nucleic acids (Zarnowski et al., 2014; Mukherjee and Chandra, 2015; Nobile and Johnson, 2015; Gulati and Nobile, 2016). These structural differences between *C. auris* and *C. albicans* biofilms could influence the efficacies of photodynamic therapy by affecting the uptake of photosensitizing compounds and the traversal of visible lights throughout the biofilm architecture (Larkin et al., 2017). In addition, differences in cell wall composition between *C. auris* and *C. albicans* cells could also impact how visible lights and photosensitizing compounds interact with the cell wall and thus impact the antibiofilm effectiveness of photodynamic therapy. The *C. auris* cell wall, for example, contains distinct cell surface mannans that are absent from the *C. albicans* cell wall as well as elevated chitin levels relative to the *C. albicans* cell wall (Navarro-arias et al., 2019; Zamith-Miranda et al., 2019; Yan et al., 2020).

Since antimicrobial photodynamic therapy relies on the localized production of ROS to cause oxidation of microbial lipids, proteins, and carbohydrates, it is likely to have broad-spectrum antimicrobial activity against many different microorganisms (Jockusch et al., 1996; Hamblin and Hasan, 2004; Maisch, 2015; Carrera et al., 2016). Indeed, there is evidence in the literature to suggest that photodynamic therapy is effective at killing a wide range of microorganisms, including pathogenic gram-positive and gram-negative bacteria, protozoa, fungi, and even viruses (Gardlo et al., 2003; Wainwright, 2004; Marotti et al., 2009; Kharkwal et al., 2011; Lyon et al., 2011; De Lucca et al., 2012; Teixeira et al., 2015). In fact, in the current COVID-19 pandemic, antimicrobial photodynamic therapy has been suggested as a potential therapeutic strategy to use against COVID-19 infections (Wiehe et al., 2019; Almeida et al., 2020; Dias and Bagnato, 2020). Consistent with this idea, one recent study demonstrated that red light in combination with photosensitizing compounds was effective at inhibiting SARS-CoV-2 viral replication within mammalian Vero E6 cells (Svyatchenko et al., 2021). Given that the prevalence of *C. auris-*SARS-CoV-2 coinfections have been increasing throughout the COVID-19 pandemic and that there is evidence to suggest that photodynamic therapy could be effective against *C. auris* and SARS-CoV-2 infections individually, photodynamic therapy could be a promising therapeutic strategy to consider for these as well as other coinfections in the clinic.

Recently, pan-resistant clinical isolates of *C. auris* that are resistant to all three of the major classes of antifungal drugs available to treat invasive fungal infections in humans have been reported in several countries, including the United States (Ostrowsky et al., 2020). Despite the emergence of these pan-resistant isolates, antifungal drugs remain the most commonly used treatment for *C. auris* infections (Cortegiani et al., 2018; Spivak and Hanson, 2018). Based on our findings as well as numerous findings in the literature on the effectiveness of antimicrobial photodynamic therapies against a multitude of pathogenic microorganisms across phylogenetic kingdoms, we believe that photodynamic therapy could be a valuable therapeutic strategy that should be explored further for use against *C. auris* infections. In the context of *C. auris* infections, there are at least three major shortcomings of traditional antifungal drug therapies that could be overcome by using photodynamic therapy. First, the development of antifungal drug resistance after exposure to antifungal drugs has the potential to render antifungal drug treatments completely ineffective against fungal infections. This is frequently observed in the context of *C. auris* infections, and in fact, the majority of *C. auris* clinical isolates are resistant to at least one antifungal drug class (Lockhart et al., 2017; Cortegiani et al., 2018; Forsberg et al., 2019). Since photodynamic therapy leads to the production of ROS that broadly affect numerous microbial targets simultaneously, it is unlikely that *C. auris* resistance to photodynamic therapy could be developed. Second, antifungal drugs, like the polyenes, are known to cause substantial toxicities in humans and are usually administered systemwide (Roemer and Krysan, 2014). The non-toxic photosensitizing compounds and the visible lights used during the administration of photodynamic therapy pose little, if any, toxicity concerns to humans (Hamblin and Hasan, 2004; Wainwright et al., 2017). Additionally, photodynamic therapy can be spatially confined to an area of interest, thus limiting unnecessary exposure of human cells to the treatment. Third, almost all existing antimicrobial drugs function by targeting microbial metabolic processes, and therefore require that the cells are metabolically active (Kuhn et al., 2002; Bojsen et al., 2014; Stokes et al., 2019). This necessity, especially within heterogeneous microbial cell populations, can cause substantial inconsistencies in effectiveness of the antimicrobial drug. This is particularly true in the context of biofilms, where different cell populations are present throughout the biofilm architecture with varying levels of metabolic activity (Wimpenny et al., 2000; Mah and O’Toole, 2001; Taff et al., 2013). Additionally, persister cells, defined as metabolically dormant phenotypic cell variants, are markedly difficult to eradicate within mature biofilms using traditional antimicrobial drugs (Stewart, 2002; Lewis, 2010; Taff et al., 2013; Fox et al., 2015). *C. auris* biofilms, in particular, are notorious for displaying low susceptibilities to existing antifungal drugs, including caspofungin and amphotericin B, which is likely the result of, at least in part, cell heterogeneity within *C. auris* biofilms (Sherry et al., 2017; Kean et al., 2018). The effectiveness of photodynamic therapy does not require that microbial cells are metabolically active; indeed, there are studies that have found that photodynamic therapy can be effective against bacterial persister cells (Hamblin and Hasan, 2004; Oppezzo and Forte Giacobone, 2018).

In summary, our results suggest that photodynamic therapy is highly effective at inhibiting *C. auris* biofilm formation and at disrupting mature *C. auris* biofilms *in vitro*. Given that our antifungal drug arsenal is extremely limited and that pan-resistant *C. auris* isolates have been emerging, new therapeutic strategies effective against *C. auris* are urgently needed. Our work suggests that photodynamic therapy could be a clinically viable option in combating *C. auris* infections that should be explored further.

## Data Availability Statement

The original contributions presented in the study are included in the article/**Supplementary Material**. Further inquiries can be directed to the corresponding author.

## Author Contributions

Conceptualization PB and CN. Methodology PB and CN. Validation PB and CN. Formal Analysis PB. Investigation PB. Resources, CN. Data Curation, PB. Writing – Original Draft Preparation, PB. Writing – Review & Editing, PB and CN. Visualization, PB. Supervision, CN. Project Administration, CN. Funding Acquisition, CN. All authors contributed to the article and approved the submitted version.

## Funding

This work was supported by the National Institutes of Health (NIH) National Institute of General Medical Sciences (NIGMS) award R35GM124594, and by the Kamangar family in the form of an endowed chair to CN. The funders had no role in the study design, data collection and interpretation, or the decision to submit the work for publication.

## Conflict of Interest

CN is a cofounder of BioSynesis, Inc., a company developing diagnostics and therapeutics for biofilm infections.

The remaining author declares that the research was conducted in the absence of any commercial or financial relationships that could be construed as a potential conflict of interest.

## Publisher’s Note

All claims expressed in this article are solely those of the authors and do not necessarily represent those of their affiliated organizations, or those of the publisher, the editors and the reviewers. Any product that may be evaluated in this article, or claim that may be made by its manufacturer, is not guaranteed or endorsed by the publisher.
