# Supplementary material for: Photodynamic Therapy Is Effective Against Candida auris Biofilms
Source: Front Cell Infect Microbiol. 2021 Sep 3;11:713092. doi: 10.3389/fcimb.2021.713092 (PMC8446617; doi:10.3389/fcimb.2021.713092)
Supplement: Supplementary file 1 [file DataSheet_1.pdf]

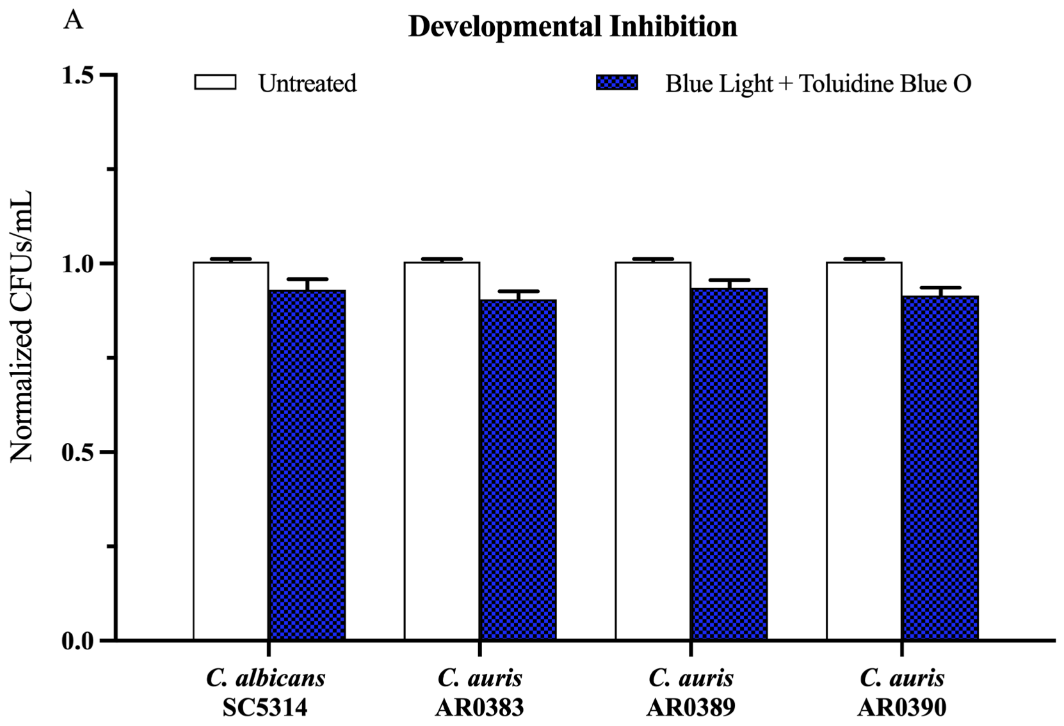

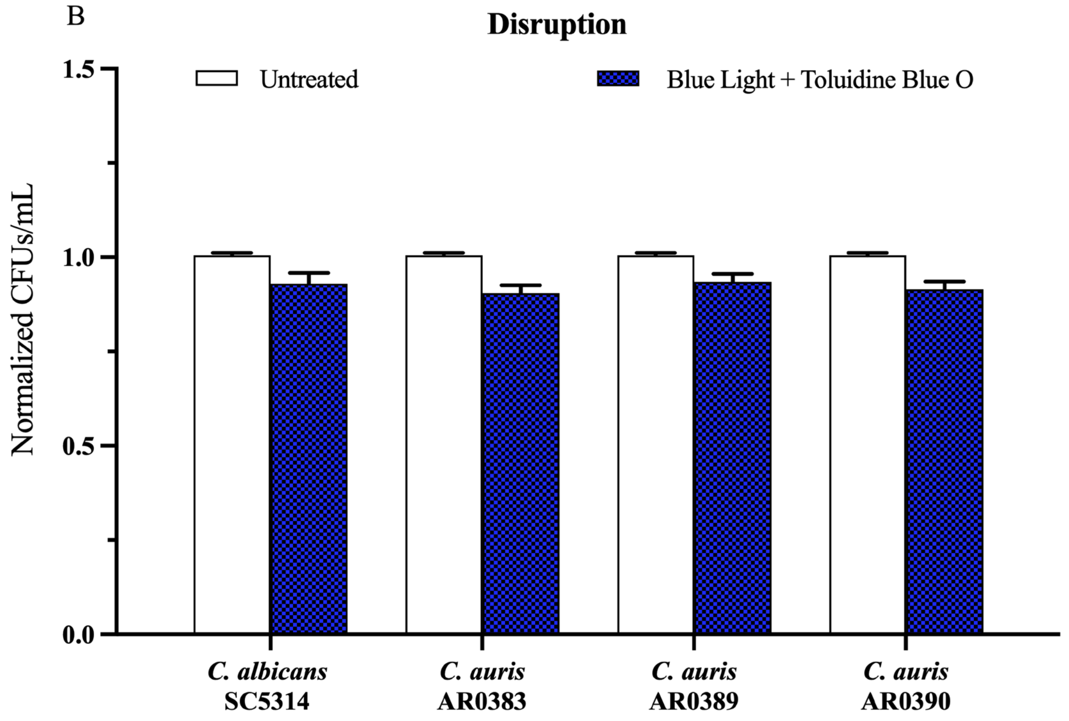

Figure S1. Blue visible light in combination with the photosensitizing compound toluidine blue O is ineffective against *C. auris* biofilms after a shortened (90 min) exposure. *C albicans*

(SC5314) and *C. auris* (AR0383, AR0389, and AR0390) biofilms were exposed to blue visible light with and without the photosensitizing compound toluidine blue O for 90 min as indicated in the (A) developmental inhibition, and (B) disruption biofilm assays. An untreated control (Untreated) and blue light in combination with the photosensitizing compound toluidine blue O (Blue Light + Toluidine Blue O) are shown. CFUs/mL were counted to determine viable cell counts at the end of each of the biofilm assays. Standard deviations are shown for each sample (n=3). The average CFUs/mL of the untreated control samples for each assay were normalized to 1.

Developmental Inhibition

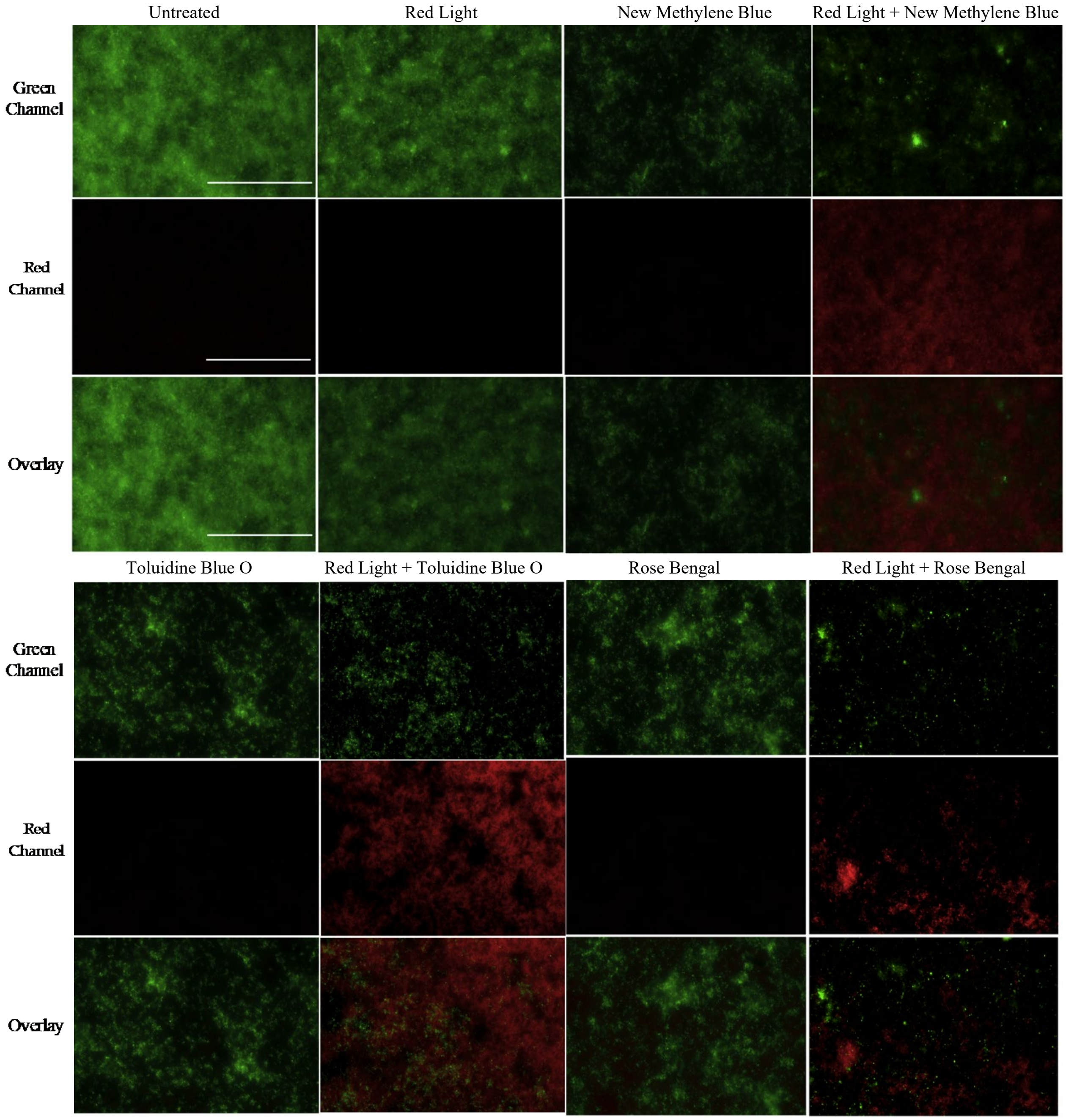

Figure S2. Red visible light in combination with photosensitizing compounds is effective at reducing the cell viability of *C. auris* biofilms in the developmental inhibition biofilm assay. The viability of *C. auris* (AR0383) biofilms was assessed using the LIVE/DEAD *Bac*Light viability kit, where green fluorescence indicates live cells, and red fluorescence indicates dead cells. The samples were imaged by fluorescence microscopy at 20X magnification with a green laser (GFP/green channel) shown in the top panels, a red laser (Texas Red/red channel) shown in

the middle panels, and overlayed shown in the bottom panels for each set of images. Representative images are shown for the untreated control (Untreated), red light alone (Red Light), new methylene blue photosensitizing compound alone (New Methylene Blue), red light in combination with new methylene blue photosensitizing compound (Red Light + New Methylene Blue), toluidine blue O photosensitizing compound alone (Toluidine Blue O), red light in combination with toluidine blue O photosensitizing compound (Red Light + Toluidine Blue O), rose bengal photosensitizing compound alone (Rose Bengal) and red light in combination with rose bengal photosensitizing compound (Red Light + Rose Bengal). Scale bars represent 200μm.

Disruption

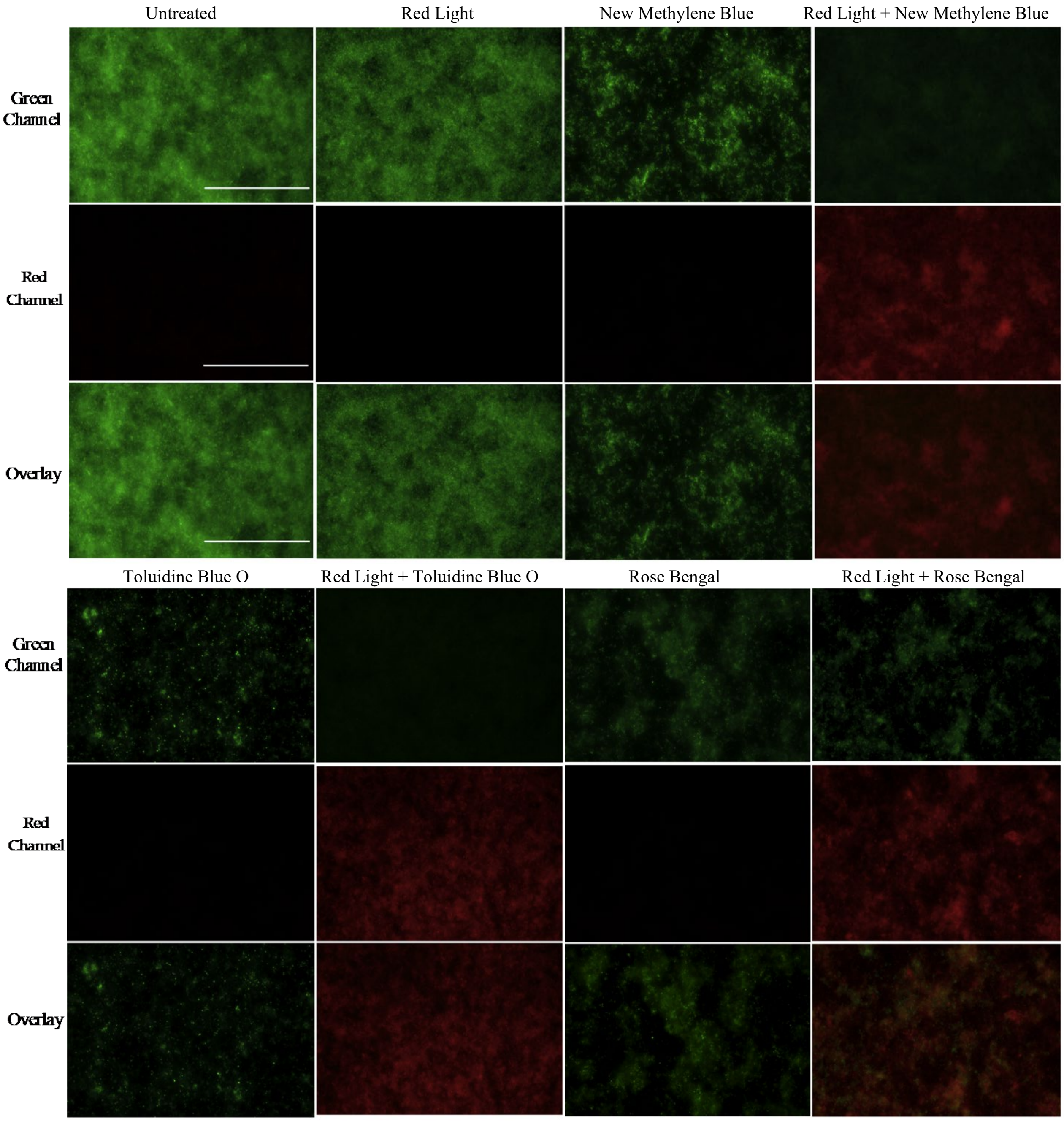

Figure S3. Red visible light in combination with photosensitizing compounds is effective at reducing the cell viability of *C. auris* biofilms in the disruption biofilm assay. The viability of *C. auris* (AR0383) biofilms was assessed using the LIVE/DEAD *Bac*Light viability kit, where green fluorescence indicates live cells, and red fluorescence indicates dead cells. The samples were imaged by fluorescence microscopy at 20X magnification with a green laser (GFP/green channel) shown in the top panels, a red laser (Texas Red/red channel) shown in the middle panels, and

overlayed shown in the bottom panels for each set of images. Representative images are shown for the untreated control (Untreated), red light alone (Red Light), new methylene blue photosensitizing compound alone (New Methylene Blue), red light in combination with new methylene blue photosensitizing compound (Red Light + New Methylene Blue), toluidine blue O photosensitizing compound alone (Toluidine Blue O), red light in combination with toluidine blue O photosensitizing compound (Red Light + Toluidine Blue O), rose bengal photosensitizing compound alone (Rose Bengal) and red light in combination with rose bengal photosensitizing compound (Red Light + Rose Bengal). Scale bars represent 200μm.

Developmental Inhibition

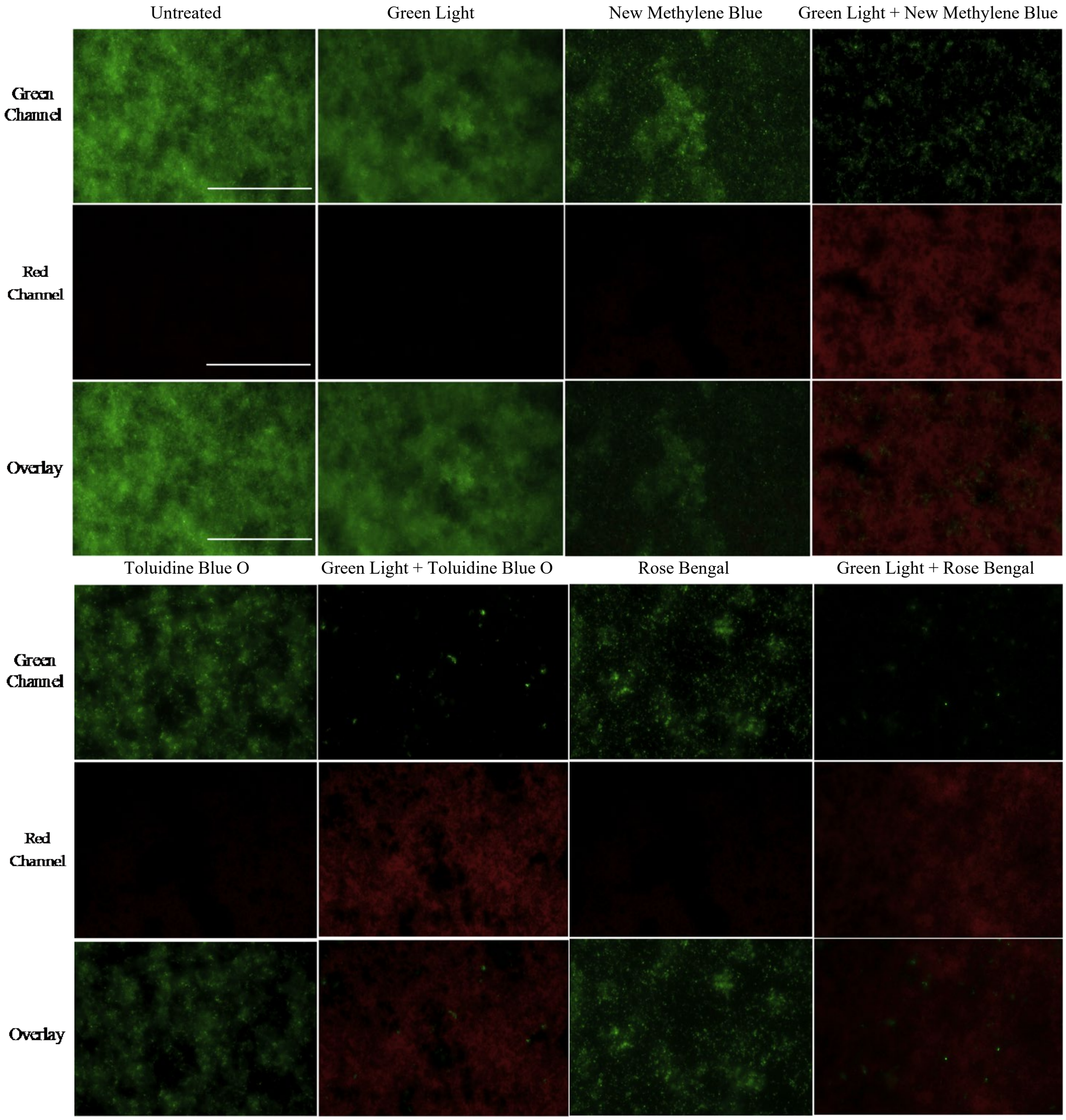

Figure S4. Green visible light in combination with photosensitizing compounds is effective at reducing the cell viability of *C. auris* biofilms in the developmental inhibition biofilm assay. The viability of *C. auris* (AR0383) biofilms was assessed using the LIVE/DEAD *Bac*Light viability kit, where green fluorescence indicates live cells, and red fluorescence indicates dead cells. The samples were imaged by fluorescence microscopy at 20X magnification with a green laser (GFP/green channel) shown in the top panels, a red laser (Texas Red/red channel) shown in the middle panels, and overlayed shown in the bottom panels for each set of images. Representative

images are shown for the untreated control (Untreated), green light alone (Green Light), new methylene blue photosensitizing compound alone (New Methylene Blue), green light in combination with new methylene blue photosensitizing compound (Green Light + New Methylene Blue), toluidine blue O photosensitizing compound alone (Toluidine Blue O), green light in combination with toluidine blue O photosensitizing compound (Green Light + Toluidine Blue O), rose bengal photosensitizing compound alone (Rose Bengal) and green light in combination with rose bengal photosensitizing compound (Green Light + Rose Bengal). Scale bars represent 200µm.

Developmental Inhibition

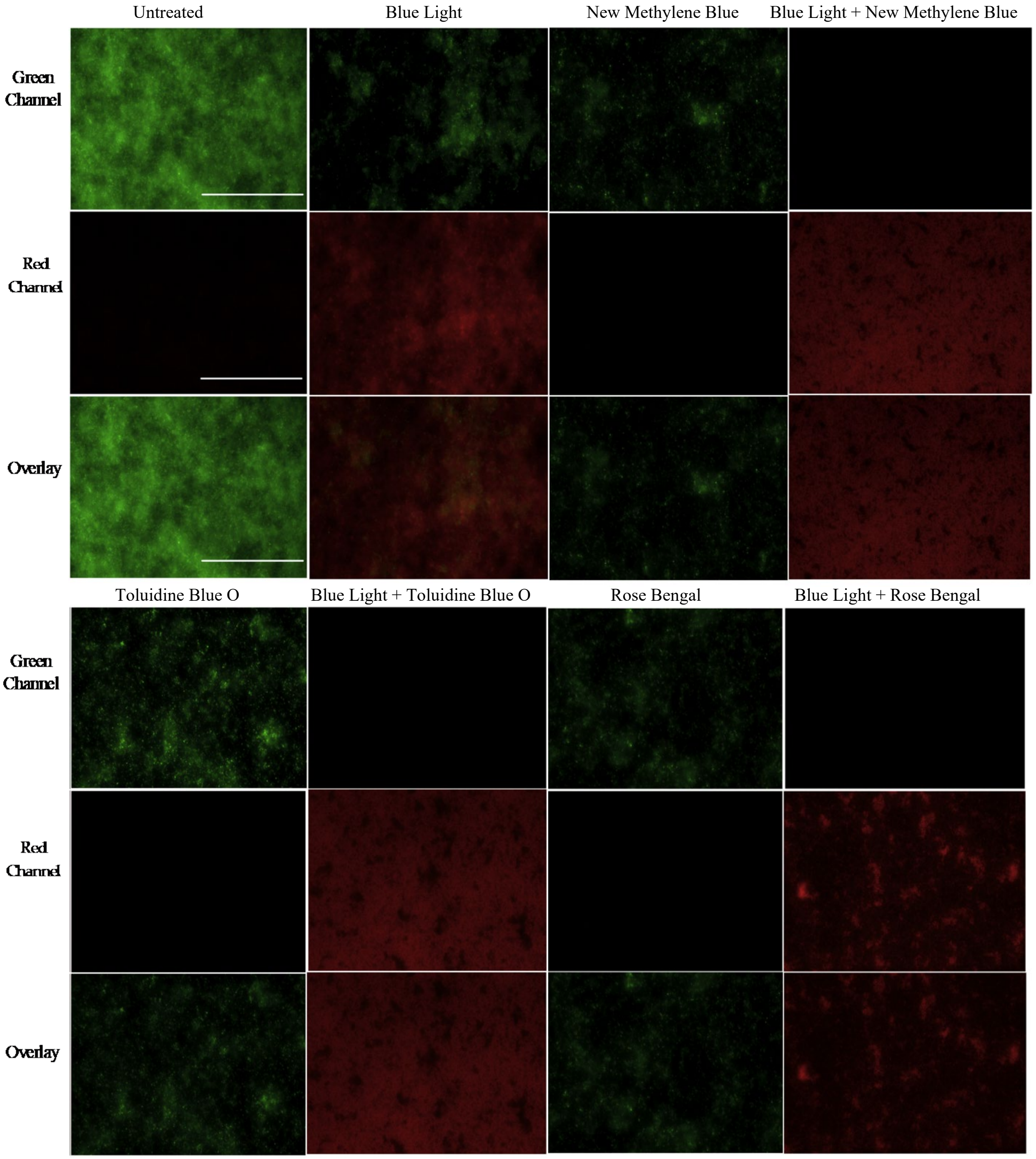

Figure S5. Blue visible light in combination with photosensitizing compounds is effective at reducing the cell viability of *C. auris* biofilms in the developmental inhibition biofilm assay. The viability of *C. auris* (AR0383) biofilms was assessed using the LIVE/DEAD *Bac*Light viability kit, where green fluorescence indicates live cells, and red fluorescence indicates dead

cells. The samples were imaged by fluorescence microscopy at 20X magnification with a green laser (GFP/green channel) shown in the top panels, a red laser (Texas Red/red channel) shown in the middle panels, and overlayed shown in the bottom panels for each set of images. Representative images are shown for the untreated control (Untreated), blue light alone (Blue Light), new methylene blue photosensitizing compound alone (New Methylene Blue), blue light in combination with new methylene blue photosensitizing compound (Blue Light + New Methylene Blue), toluidine blue O photosensitizing compound alone (Toluidine Blue O), blue light in combination with toluidine blue O photosensitizing compound (Blue Light + Toluidine Blue O), rose bengal photosensitizing compound alone (Rose Bengal) and blue light in combination with rose bengal photosensitizing compound (Blue Light + Rose Bengal). Scale bars represent 200μm.

Disruption

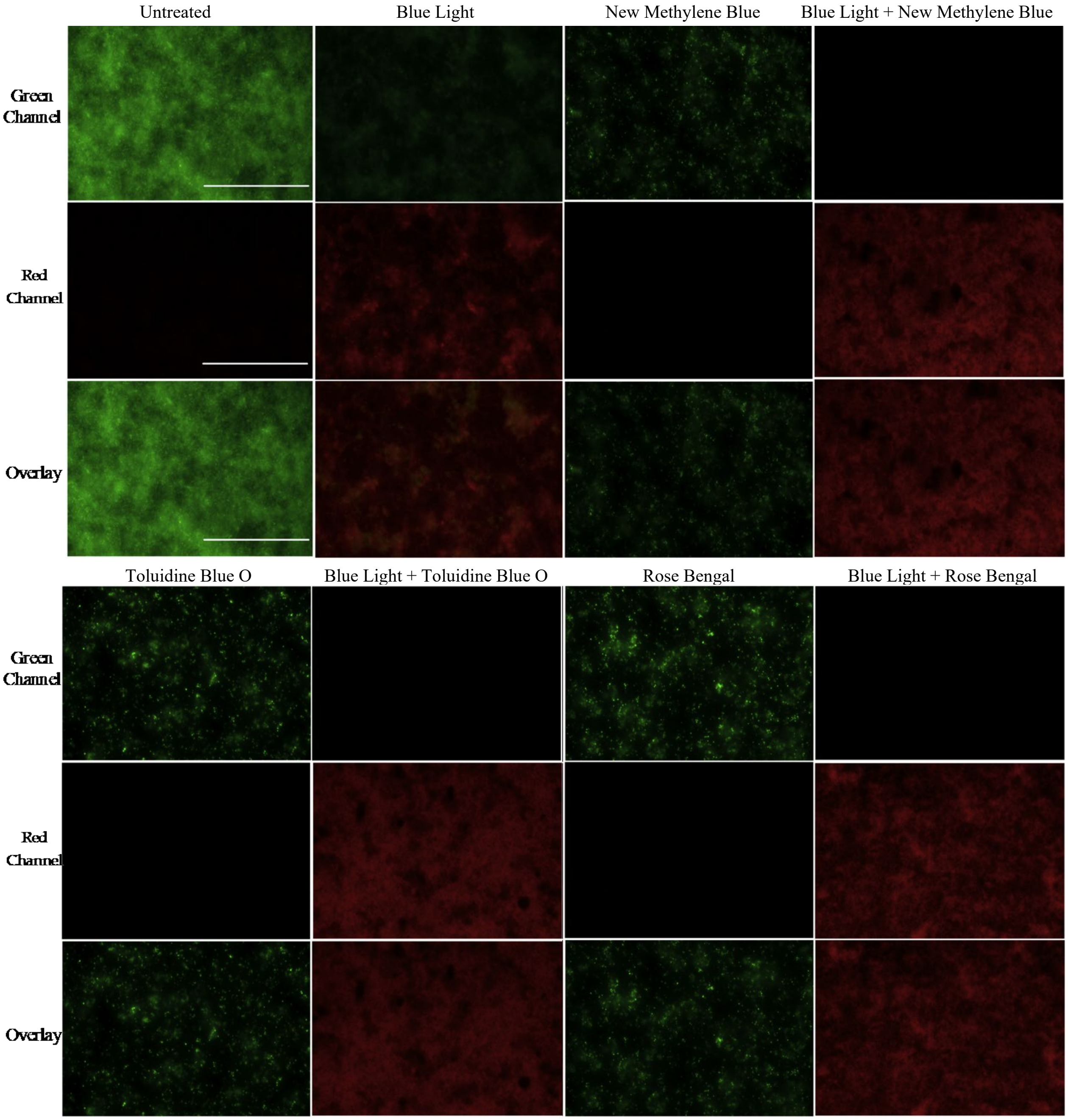

Figure S6. Blue visible light in combination with photosensitizing compounds is effective at reducing the cell viability of *C. auris* biofilms in the disruption biofilm assay. The viability of *C. auris* (AR0383) biofilms was assessed using the LIVE/DEAD *Bac*Light viability kit, where green fluorescence indicates live cells, and red fluorescence indicates dead cells. The samples were imaged by fluorescence microscopy at 20X magnification with a green laser (GFP/green channel) shown in the top panels, a red laser (Texas Red/red channel) shown in the middle panels, and overlayed shown in the bottom panels for each set of images. Representative images are shown for

the untreated control (Untreated), blue light alone (Blue Light), new methylene blue photosensitizing compound alone (New Methylene Blue), blue light in combination with new methylene blue photosensitizing compound (Blue Light + New Methylene Blue), toluidine blue O photosensitizing compound alone (Toluidine Blue O), blue light in combination with toluidine blue O photosensitizing compound (Blue Light + Toluidine Blue O), rose bengal photosensitizing compound alone (Rose Bengal) and blue light in combination with rose bengal photosensitizing compound (Blue Light + Rose Bengal). Scale bars represent 200μm.

Developmental Inhibition

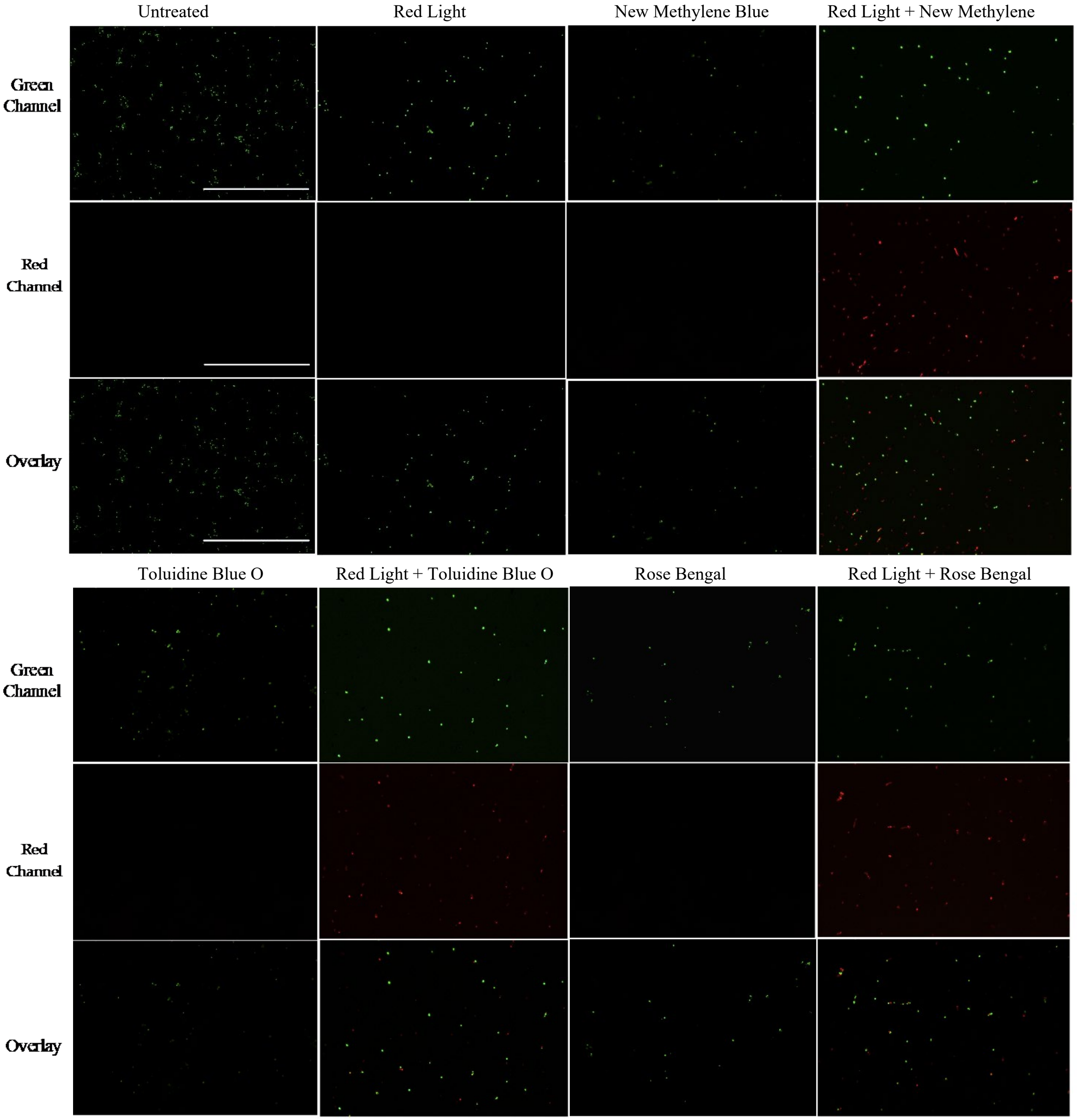

Figure S7. Red visible light in combination with photosensitizing compounds is effective at reducing the cell viability of cells resuspended from C. *auris* biofilms in the developmental inhibition biofilm assay. The viability of *C. auris* (AR0383) cells resuspended from biofilms was assessed using the LIVE/DEAD *Bac*Light viability kit, where green fluorescence indicates live cells, and red fluorescence indicates dead cells. The samples were imaged by fluorescence microscopy at 20X magnification with a green laser (GFP/green channel) shown in the top panels, a red laser (Texas Red/red channel) shown in the middle panels, and overlayed shown in the bottom panels for each set of images. Representative images are shown for the untreated control

(Untreated), red light alone (Red Light), new methylene blue photosensitizing compound alone (New Methylene Blue), red light in combination with new methylene blue photosensitizing compound (Red Light + New Methylene Blue), toluidine blue O photosensitizing compound alone (Toluidine Blue O), red light in combination with toluidine blue O photosensitizing compound (Red Light + Toluidine Blue O), rose bengal photosensitizing compound alone (Rose Bengal) and red light in combination with rose bengal photosensitizing compound (Red Light + Rose Bengal). Scale bars represent 200µm.

Disruption

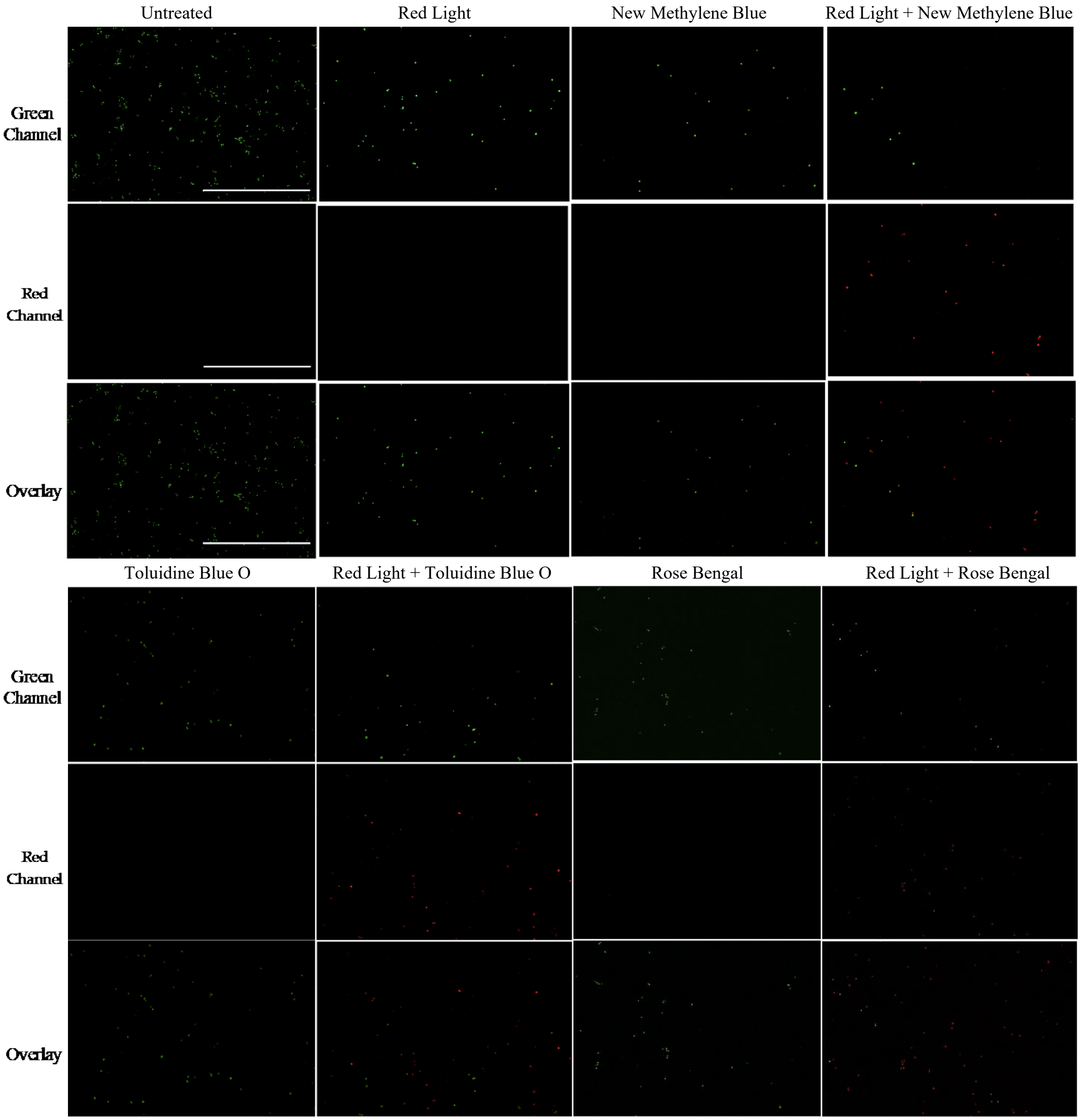

Figure S8. Red visible light in combination with photosensitizing compounds is effective at reducing the cell viability of cells resuspended from C. *auris* biofilms in the disruption biofilm assay. The viability of *C. auris* (AR0383) cells resuspended from biofilms was assessed using the LIVE/DEAD *Bac*Light viability kit, where green fluorescence indicates live cells, and red fluorescence indicates dead cells. The samples were imaged by fluorescence microscopy at 20X magnification with a green laser (GFP/green channel) shown in the top panels, a red laser (Texas Red/red channel) shown in the middle panels, and overlayed shown in the bottom panels for each

set of images. Representative images are shown for the untreated control (Untreated), red light alone (Red Light), new methylene blue photosensitizing compound alone (New Methylene Blue), red light in combination with new methylene blue photosensitizing compound (Red Light + New Methylene Blue), toluidine blue O photosensitizing compound alone (Toluidine Blue O), red light in combination with toluidine blue O photosensitizing compound (Red Light + Toluidine Blue O), rose bengal photosensitizing compound alone (Rose Bengal) and red light in combination with rose bengal photosensitizing compound (Red Light + Rose Bengal). Scale bars represent 200μm.

Developmental Inhibition

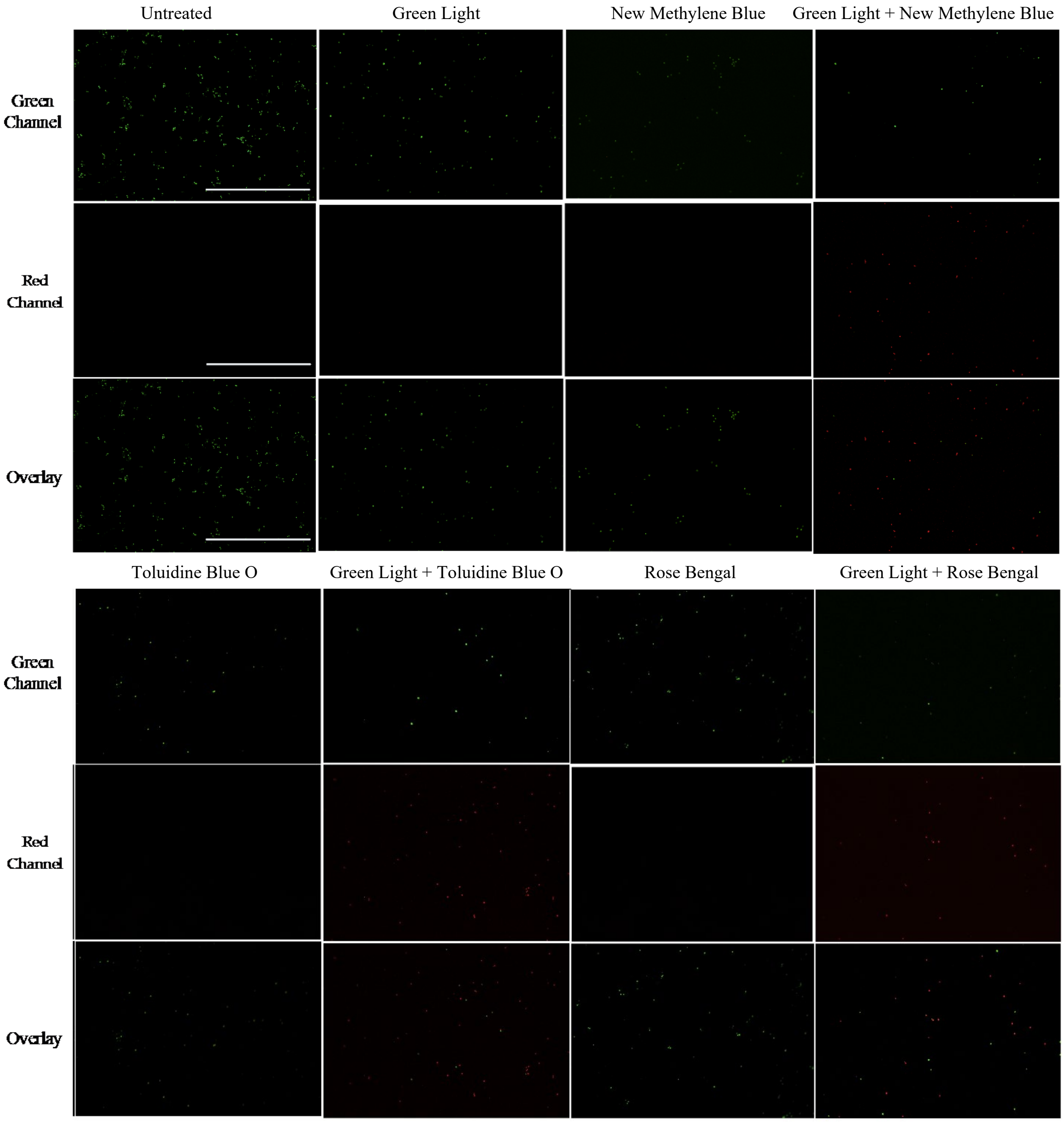

Figure S9. Green visible light in combination with photosensitizing compounds is effective at reducing the cell viability of cells resuspended from C. *auris* biofilms in the developmental inhibition biofilm assay. The viability of *C. auris* (AR0383) cells resuspended from biofilms was assessed using LIVE/DEAD *Bac*Light viability kit, where green fluorescence indicates live cells, and red fluorescence indicates dead cells. The samples were imaged by fluorescence microscopy at 20X magnification with a green laser (GFP/green channel) shown in the top panels, a red laser

(Texas Red/red channel) shown in the middle panels, and overlayed shown in the bottom panels for each set of images. Representative images are shown for the untreated control (Untreated), green light alone (Green Light), new methylene blue photosensitizing compound alone (New Methylene Blue), green light in combination with new methylene blue photosensitizing compound (Green Light + New Methylene Blue), toluidine blue O photosensitizing compound alone (Toluidine Blue O), green light in combination with toluidine blue O photosensitizing compound (Green Light + Toluidine Blue O), rose bengal photosensitizing compound alone (Rose Bengal) and green light in combination with rose bengal photosensitizing compound (Green Light + Rose Bengal). Scale bars represent 200μm.

Developmental Inhibition

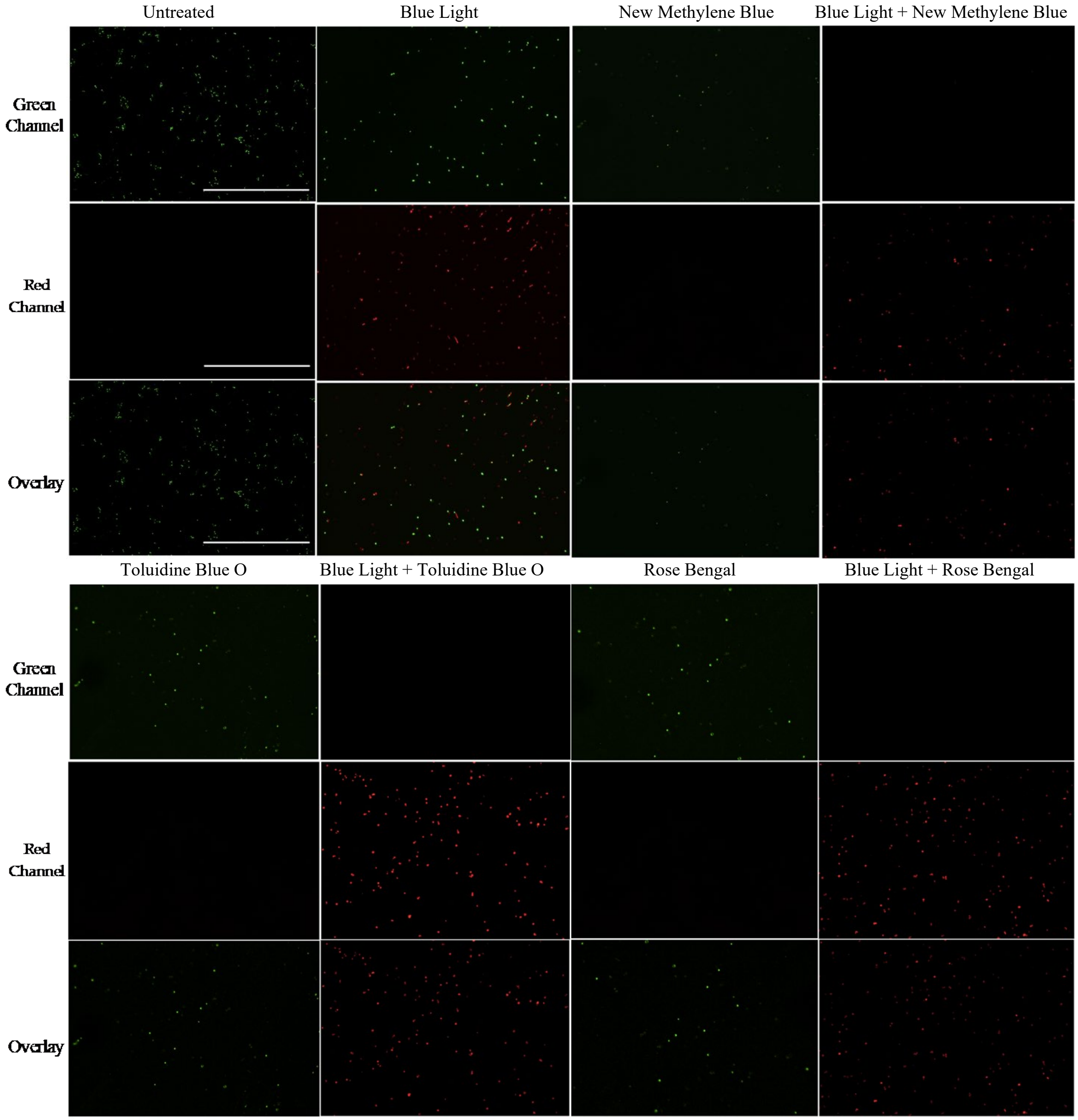

Figure S10. Blue visible light in combination with photosensitizing compounds is effective at reducing the cell viability of cells resuspended from C. *auris* biofilms in the developmental inhibition biofilm assay. The viability of *C. auris* (AR0383) cells resuspended from biofilms was assessed using the LIVE/DEAD *Bac*Light viability kit, where green fluorescence indicates live cells, and red fluorescence indicates dead cells. The samples were imaged by fluorescence microscopy at 20X magnification with a green laser (GFP/green channel) shown in the top panels,

a red laser (Texas Red/red channel) shown in the middle panels, and overlayed shown in the bottom panels. Representative images are shown for the untreated control (Untreated), blue light alone (Blue Light), new methylene blue photosensitizing compound alone (New Methylene Blue), blue light in combination with new methylene blue photosensitizing compound (Blue Light + New Methylene Blue), toluidine blue O photosensitizing compound alone (Toluidine Blue O), blue light in combination with toluidine blue O photosensitizing compound (Blue Light + Toluidine Blue O), rose bengal photosensitizing compound alone (Rose Bengal) and blue light in combination with rose bengal photosensitizing compound (Blue Light + Rose Bengal). Scale bars represent 200μm.

Disruption

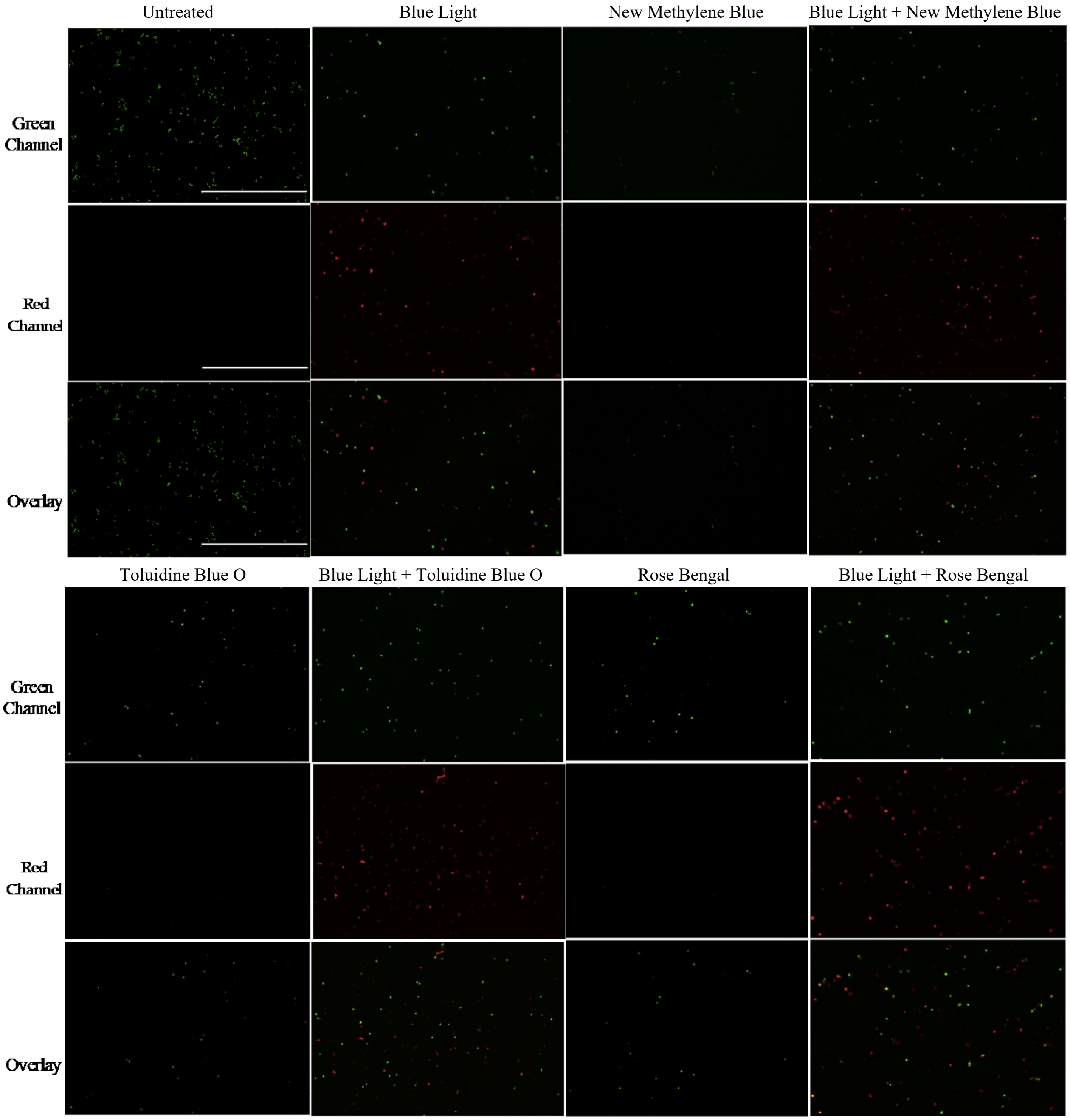

Figure S11. Blue visible light in combination with photosensitizing compounds is effective at reducing the cell viability of cells resuspended from C. *auris* biofilms in the disruption biofilm assay. The viability of *C. auris* (AR0383) cells resuspended from biofilms was assessed using the LIVE/DEAD *Bac*Light viability kit, where green fluorescence indicates live cells, and red fluorescence indicates dead cells. The samples were imaged by fluorescence microscopy at 20X magnification with a green laser (GFP/green channel) shown in the top panels, a red laser (Texas

Red/red channel) shown in the middle panels, and overlayed shown in the bottom panels for each set of images. Representative images are shown for the untreated control (Untreated), blue light alone (Blue Light), new methylene blue photosensitizing compound alone (New Methylene Blue), blue light in combination with new methylene blue photosensitizing compound (Blue Light + New Methylene Blue), toluidine blue O photosensitizing compound alone (Toluidine Blue O), blue light in combination with toluidine blue O photosensitizing compound (Blue Light + Toluidine Blue O), rose bengal photosensitizing compound alone (Rose Bengal) and blue light in combination with rose bengal photosensitizing compound (Blue Light + Rose Bengal). Scale bars represent 200μm.

Table S1. Reported MICs for the *C. auris* strains used in this study.

	AR Bank Isolate, Clade, and MICs (μg/mL) [#]		
Antifungal drugs	AR0383 (South Africa)	AR0389 (South Asia)	AR0390 (South Asia)
Amphotericin B	0.38	4	4
Fluconazole	128	256	>256
Caspofungin	0.25	0.5	0.5

[#]MICs were reported in Lockhart et al., 2017; and https://www.cdc.gov/fungal/candida-auris/c-auris-antifungal.html/; accessed on 05/07/2021.
